# A Precarious Path to Partnership? The Moderating Effects of Labour Market Regulations on the Relationship Between Unstable Employment and Union Formation in Europe

**DOI:** 10.1007/s10680-024-09698-3

**Published:** 2024-03-29

**Authors:** Chen-Hao Hsu, Henriette Engelhardt

**Affiliations:** 1 Institute for Sociology, University of Bamberg, Bamberg, Germany; 2 The State Institute for Family Research (ifb) at the University of Bamberg, Bamberg, Germany

**Keywords:** Union formation, Unstable employment, Labour market regulations, Institutions, Multilevel analysis, Country fixed effects models

## Abstract

Rising employment uncertainty featured by higher risks of being temporarily employed or unemployed is often seen as the driving force behind delayed and declined partnering in Western countries. However, such an employment–partnering relationship is contextualized by labour market institutions and thus could diverge across countries over time. This paper aims to investigate how country-level variations in labour market regulations moderate individual-level effects of unstable employment on union formation, including the transitions into marriage or cohabitation unions. Using comparative panel data for 26 countries from the European Union Statistics on Income and Living Conditions (the years 2010–2019), our multilevel fixed effects models showed that temporary employment and unemployment negatively affected the probability of union formation for single women and men in Europe. Moreover, the negative relationship between unstable employment and union formation was reinforced when labour market reforms were stimulating insider–outsider segregations or decreasing welfare provisions. Specifically, stricter employment protection legislations and higher coverage rates of collective bargaining agreements could reinforce the negative effects of temporary employment and unemployment on union formation, while more generous provisions of unemployment benefits could buffer such negative effects.

**Supplementary Information:**

The online version contains supplementary material available at 10.1007/s10680-024-09698-3.

## Introduction

Since the 1990s, labour market reforms in many European countries have dramatically changed individuals' working conditions and life experiences (Esping-Andersen & Regini, 2000; Kalleberg, 2018). Compared to workers of older generations, who mostly have stable employment careers in permanent positions, workers nowadays experience higher risks of unemployment and temporary employment by fixed-term contracts in their lives (Gallie & Paugam, 2000; Latner, 2022). Under the trend of rising employment uncertainty (Blossfeld et al., 2005; Kreyenfeld et al., 2012), individuals’ work and family life courses have also experienced remarkable changes in the past two decades (Balbo et al., 2013). To tackle the underlying mechanisms behind the ongoing demographic transition towards delayed and declined partnering and fertility, this paper studied how people’s unstable employment experiences, including temporary employment and unemployment, affect their union formation (i.e. the transition into marriage or cohabitating household unions).

Several studies found that unemployment or temporary employment may reduce individuals’ risks of getting married or cohabiting (Bolano & Vignoli, 2021; de Lange et al., 2014; Kalmijn, 2011; Müller & Dräger, 2019; van Wijk et al., 2021). Although these studies provided valuable insights, they mostly relied on single-country case analyses and underemphasized the institutional factors behind the observed relationships between work and union formation. In contrast, research on the social consequences of changing labour markets usually utilized cross-national comparative designs to highlight the role of institutions in eliminating or escalating social inequalities across different employment groups (Esping-Andersen & Regini, 2000). However, most studies focused on labour market policies’ impacts on economic inequalities such as income and job insecurity (Balz, 2017; Barbieri & Cutuli, 2016; Högberg et al., 2019); only a few discussed how inequalities in other life course dimensions such as childbirth and partnering were influenced by different institutional settings (Barbieri et al., 2015; Hsu, 2023; Karabchuk, 2020; Scherer, 2009).

In this paper, we highlighted the role of labour market regulations in shaping the structural conditions of partnership behaviours, arguing that they could either mitigate or intensify the differentials in union formation probabilities across employment groups. For example, stricter employment protection laws may arouse labour market segregation between the “core” and the “peripheral” labour forces and sharpen the social inequality in income and employment securities between these two groups (Barbieri, 2009). As a result, having stricter employment protection laws at the country level might enlarge the disadvantage of the unemployed or the temporarily employed in finding a partner or starting a family.

In summary, this study investigated two central questions: (1) How do people’s unstable employment situations including temporary employment and unemployment affect their likelihood of union formation? And (2) to what extent are such effects moderated by country-level variations in labour market regulations, including the strictness of the employment protection laws for both regular and temporary workers, the coverage rate of collective bargaining agreements, and the generosity of unemployment benefits? We also discussed the gendered impact of unstable employment on union formation and the gendering role of labour market contexts in shaping such work–family relationships.

Our empirical analyses were based on comparative microdata from the European Union Statistics on Income and Living Conditions (EU-SILC), collected during the years 2010–2019 across 26 European countries. Leveraging the longitudinal nature of the data, we applied country fixed effects and slopes multilevel models (Giesselmann & Schmidt-Catran, 2019) to provide more precise estimations of policy moderating effects. Our findings not only contribute to theoretical literature by highlighting institutional factors behind the changing relationship between work and family formation (Blossfeld et al., 2005) but also add crucial policy implications to the debate on labour market “flexicurity” and its social consequences in post-Great Recession Europe (Eichhorst et al., 2017; Viebrock & Clasen, 2009).

## Background

### Employment Situations, Insecurities, and Union Formation: Micro-level Theories

Compared to stable employment by permanent contract, unstable employment situations such as being unemployed or temporarily employed are usually characterized by (1) income insecurity—the difficulty in securing sufficient incomes with which to support a decent standard of living—and (2) employment insecurity—the tendency to lose jobs shortly or remain unemployed (Grotti & Scherer, 2014; Kalleberg, 2009; Olsthoorn, 2014). In family research, income and employment insecurities are key mechanisms behind delayed or decreased union formation (Ekert-Jaffe & Solaz, 2001; Kalmijn, 2011; Oppenheimer, 1988, 2003).

Due to the lack of salary, unemployment has always been a major factor of household income poverty (Haataja, 1999). Even in countries with generous welfare provisions, social insurance for the unemployed cannot entirely offset their losses of earnings (Gallie & Paugam, 2000). Among the employed, temporary employees have lower bargaining power over their wages and benefits (Gebel, 2010). Employers also have fewer incentives to provide on-the-job training for temporary workers (Forrier & Sels, 2003). Moreover, frequent employment interruptions following the end of each contract also discourage job-specific human capital accumulation for temporary employees, leading to poorer wage incomes. As a result, the unemployed and temporary employees generally have more financial problems and higher income insecurity compared to permanent employees (Gallie & Paugam, 2000; Scherer, 2009; Van Lancker, 2012).

According to the New Home Economics (Becker, 1991), income insecurity may discourage union formation through an “income effect” mechanism. Specifically, the economic theory assumes that individuals are rationally comparing the consumptions and resources—both monetary and non-monetary—that they would gain access to by partnering with a particular person (Grossbard, 2015). Sociological research also highlights the importance of financial affordability in people’s union formation, showing that individuals’ sufficient and secure income plays an important role in successful partnership matching, particularly for men (Sassler & Lichter, 2020).

Besides income insecurity, the unemployed and temporary employees face higher insecurity in their employment prospects. Research on the “scar effects” argues that unemployment experiences not only cause income constraints but also stigmatize one’s employment history, leading to long-term negative effects on subsequent earnings and career mobility (Gangl, 2004, 2006). Even after reemployment, the once unemployed may continue to perceive higher employment insecurity compared to the permanently employed (Dieckhoff, 2011; Voßemer, 2019). Among the employed, temporary workers suffer from higher employment insecurity than permanent employees (Balz, 2017). Because temporary workers’ jobs are guaranteed within a fixed term (Kalleberg, 2009), their employment prospects depend on the contract duration, the chance of another fixed-term contract renewal, and the possibility of a permanent contract transition.

Oppenheimer (2003) highlights the role of employment insecurity in delaying union formation, arguing that employment insecurity leads to uncertainty about future career paths and lifestyles. As a response, these individuals may perceive their current career stage as “immature” and choose to postpone union formation until a stable labour market position is achieved (Kalmijn, 2011). From a normative perspective, employment insecurity also disturbs assortative mating (Oppenheimer, 1988), particularly for men in a society with male-breadwinner family norms. Those who do not have a stable employment career are labelled as incapable or unattractive. Accordingly, we expect:

#### H1

*Having an unstable employment situation such as temporary employment or unemployment has a negative effect on the probability of union formation among single individuals.*

It is worth mentioning that New Home Economics and classic sociological theories usually predict a gender-asymmetric relationship between unstable employment and union formation (Sassler & Lichter, 2020). Under the conservative male-breadwinner-female-homemaker norm, men’s unstable employment has long been considered an obstacle to union formation (Oppenheimer, 2003), while women’s unstable employment used to be linked to easier partnership matching (England & Farkas, 2017). However, with the rise of gender equality and dual-earner families, recent studies showed that women’s precarious and unstable employment could discourage union formation similarly as men’s (Bolano & Vignoli, 2021; Schneider et al., 2019). Nevertheless, although women’s and men’s work–family patterns were converging in Europe (Lesnard et al., 2016), such convergence was likely driven by more women adopting traditional male employment patterns instead of more men reducing work commitment (McMunn et al., 2015). Therefore, having a stable employment career to support a family union remains a social expectation that applies primarily to men than to women.

#### H2

*The negative effect of unstable employment on union formation is stronger for men than for women.*

### Moderating Roles of Labour Market Regulations

While the above-mentioned theories predict averagely negative effects of temporary employment and unemployment on union formation, such effects may vary across countries. Previous research found that variations in macro-contexts, such as economic conditions (de Lange et al., 2014) and gender equality (Kalmijn, 2011), may partially explain the cross-national heterogeneity. In this study, we further highlight the roles of labour market regulations in shaping peoples’ employment experiences (Esping-Andersen & Regini, 2000; Gallie & Paugam, 2000). Specifically, the degree to which temporarily employed and unemployed people feel insecure about their income or employment prospects is influenced by labour market contexts (Barbieri & Cutuli, 2016; Esping-Andersen & Regini, 2000). Following the seminal work of Esping-Andersen and Regini (2000) on the social consequences of labour market deregulation, we focused on three dimensions of labour market regulations including the employment protection legislations, the collective bargaining institutions, and the unemployment welfare policies. Variations in these regulations either reinforce or attenuate the income or employment insecurities related to unstable employment situations, leading to heterogeneous relationships between unstable employment and union formation.

The first dimension is the strictness of employment protection legislations (EPL), which regulates the procedures of employee recruitment and dismissal. According to the labour market insider–outsider hypothesis, a stricter EPL at the country level may enlarge the employment “insecurity gap” between the labour market outsiders—the unemployed and temporary employees—and the insiders—permanent employees (Balz, 2017). In theory, it is important to discuss two sub-mechanisms behind such gap-widening effect by differentiating the EPL of dismissing regular workers (EPLR) from the EPL of using temporary contracts (EPLT) (Barbieri & Cutuli, 2016; OECD, 2014).

First, stricter EPLR raises the direct costs and procedure difficulties of dismissing a regular worker, which may discourage employers from hiring new workers and thus prolong the duration of unemployment and lower the risk of reemployment among the unemployed (Esping-Andersen & Regini, 2000). Because the cost of terminating a permanent contract is higher with stricter EPLR, employers also have more incentives using temporary contracts over permanent contracts to secure numerical flexibility, leading to lower chances of permanent employment transitions for temporary employees (Högberg et al., 2019).

Second, stricter EPLT constraints the usage, duration, and renewal of temporary contracts. This could raise the costs of hiring temporary workers, thus discouraging the reemployment of the unemployed in such positions as entry-level jobs. The influences of stricter EPLT on employment situations are more complex for temporary workers. On one hand, stricter EPLT prevents temporary workers from arbitrary dismissals, which buffers employment insecurity during the contract period. On the other hand, stricter EPLT may cause more frequent unemployment outflows or long-term unemployment among temporary workers because the regulated duration of temporary contracts is shorter, and multiple contract renewals become difficult (Högberg et al., 2019). Empirical findings in Europe seemed to support the primacy of the second mechanism because stricter EPLT was found to enlarge the employment insecurity gap between permanent and temporary workers (Balz, 2017).

Taken together, we argue that stricter EPLR and EPLT may both enlarge the employment insecurity gaps between labour market insiders and outsiders because the unemployed and temporary employees are more likely to be stuck in their current employment situations. Nevertheless, such reinforcement effects should be stronger for unemployment than temporary employment because the latter may become a common employment alternative when EPLR becomes stricter and is more protected than the unemployed in the context of strict EPLT.

#### H3a

*Stricter EPLR reinforces the negative effect of unstable employment on union formation.*

#### H3b

*The reinforcement effect of stricter EPLR is particularly strong in the negative relationship between unemployment and union formation.*

#### H4a

*Stricter EPLT reinforces the negative effect of unstable employment on union formation.*

#### H4b

*The reinforcement effect of stricter EPLT is particularly strong in the negative relationship between unemployment and union formation.*

The second dimension of labour market regulations deals with the degree to which wage and job qualities are settled by collective labour institutions, such as collective bargaining agreements. Although previous research has highlighted the within-sector wage-equalizing effect of higher collective bargaining coverage (CBC) rates for employees (Hayter & Weinberg, 2011), some studies also show that higher CBC has the potential to attenuate labour market dualism by marginalizing the labour market outsiders (Baranowska & Gebel, 2010; Dieckhoff et al., 2015). Because the negotiated rights and benefits covered by collective bargaining agreements only apply to those who are employed, higher CBC may further marginalize the unemployed, leading to a more negative effect of unemployment on union formation. For temporary workers, the influence of higher CBC on employment experiences is mixed. On one hand, higher CBC may provide temporary workers with stronger bargaining power over their employment wages and benefits, which could reduce wage inequalities between temporary and permanent employees (Hayter & Weinberg, 2011) and close the income insecurity gap. On the other hand, employment protections for labour market insiders are attenuated in countries with high CBC, which might preclude temporary workers from getting permanent jobs (Baranowska & Gebel, 2010) and enlarge the employment insecurity gap. While acknowledging collective agreements’ effort to eliminate wage and working condition inequalities, recent studies have shown that these agreements do not always create an inclusive workplace and may even reinforce the insider–outsider dualism because the contents of bargaining tend to overrepresent regular workers’ interests (Barton et al., 2021; Bentolila et al., 2012). Therefore, we argue that the gap-widening effect of higher CBC on the employment insecurity gap should be the dominant mechanism that escalates the negative effect of temporary employment on union formation.

#### H5a

*Higher CBC reinforces the negative effect of unstable employment on union formation.*

#### H5b

*The reinforcement effect of higher CBC is particularly strong in the negative relationship between unemployment and union formation.*

The third dimension refers to social welfares as income safety nets for the disadvantaged groups. The most employment-relevant welfare policy is the unemployment benefits (UB) system. Generous benefits for the unemployed could offset income insecurity perceived during unemployment (Voßemer, 2019), thus partially relieving the negative effects of unemployment on union formation. Generous benefits may also change the job-searching behaviours of the unemployed, permitting them to take the time to search for adequate reemployment without rushing into undermatched positions. As a result, the scar effects of unemployment on the subsequent employment and income prospects would be lower in the context of generous unemployment benefits (Gangl, 2004, 2006). For temporary employees, knowing the existence of income safety nets after ending fixed-term contracts may relieve their feelings of income insecurity. Generous benefits also reduce the employment instability of temporary workers by increasing subsequent job tenure and the probability of transitioning into a permanent job (Rebollo-Sanz & García-Pérez, 2015).

#### H6

*More generous unemployment benefits attenuates the negative effect of unstable employment on union formation.*

**Fig. 1:**
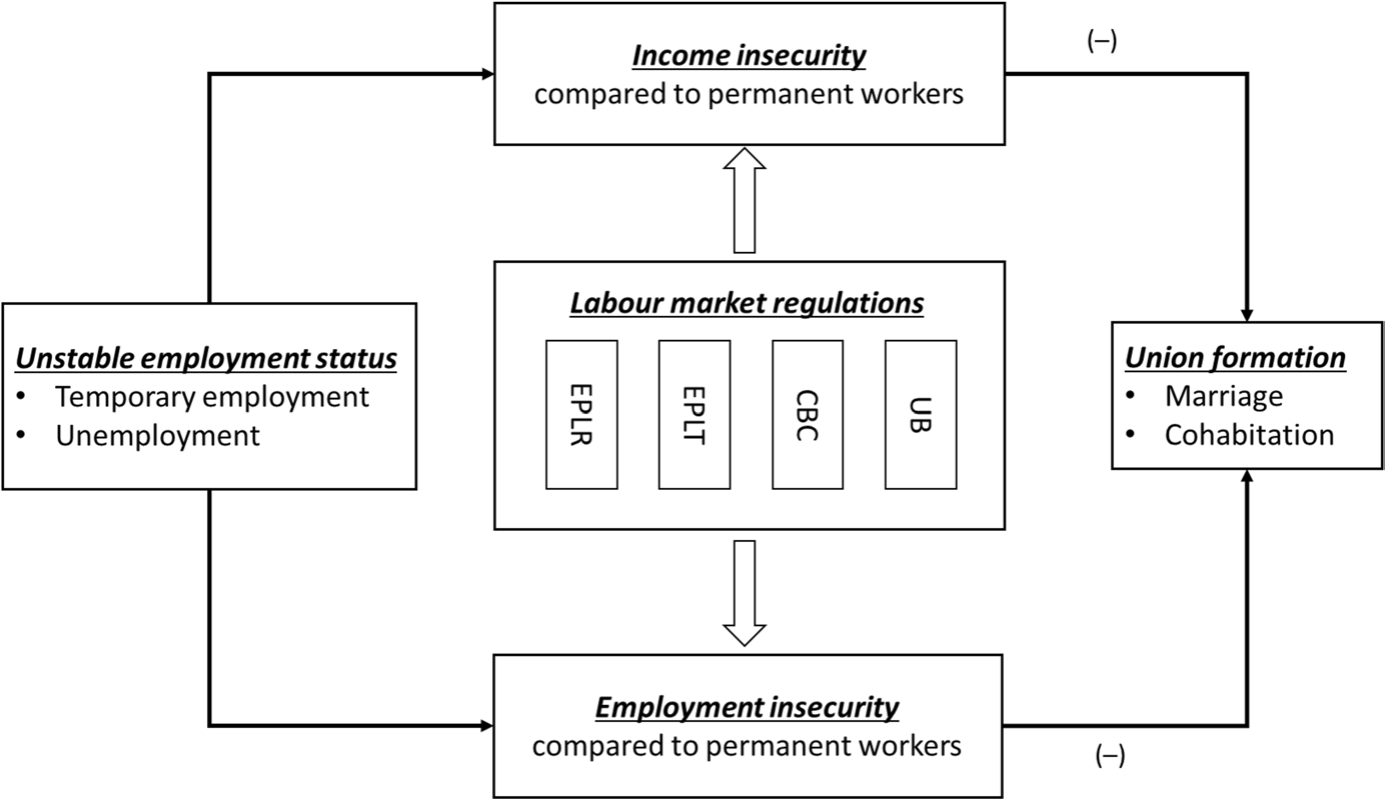
Conceptual model regarding the moderating roles of labour market regulations. Note: EPLR = “employment protection legislations of dismissing regular workers”, EPLT = “employment protection legislations of using temporary contract”, CBC = “collective bargaining agreement coverage”, UB = “unemployment benefits”

Figure 1 offers an overview of our theoretical model. We also summarized the mechanisms behind each policy moderating effects in Table S1.

Finally, labour markets are gendered institutes in which individuals’ economic behaviours are embedded in social and gender norms (Elson, 1999). Changes in labour market regulations may have gendering impacts on individuals’ union formation through interacting with the gender norms attached to specific employment status. Despite the gender convergence in life course employment patterns in many European countries (Lesnard et al., 2016), men still bear the expectation of fulfilling the primary family provider role while women’s paid work is normatively expected to be subordinated to family responsibilities (Oláh et al., 2021; Van Lancker, 2012). Although permanent employment might be the only acceptable employment situation for most men, temporary employment could still be desirable for some women, especially considering its higher flexibility to combine work with family and better income security compared to unemployment. In this regard, non-permanently employed men are more vulnerable to policy reforms that create barriers to obtaining permanent jobs, such as a stricter EPLR. On the other hand, non-permanently employed women might be more vulnerable to reforms that decrease the employability of the unemployed and the flexibility of temporary employment, such as stricter EPLT.

#### H7

*Stricter EPLR reinforces the negative effect of unstable employment on union formation especially for men.*

#### H8

*Stricter EPLT reinforces the negative effect of unstable employment on union formation especially for women.*

## Research Design

### Data and Sample

We used data at both micro- and macro-levels. Micro-level data came from the 2010–2019 EU-SILC panel data files, in which sampled individuals were interviewed annually for up to 4 years in a rotational panel design. The EU-SILC panel is a valuable source for comparative life course research because it is the only (ongoing) micro-panel dataset that features nationally representative samples across more than 30 European countries. Macro-level data regarding labour market regulations and other indices were assembled from multiple sources including the OECD, Eurostat, and the KOF Globalization Index. We combined the macro-data with all available waves of EU-SILC data during the years 2010–2019. By excluding country-waves where macro-data were unavailable, our final sample covered 215 country-waves from 26 countries. Bulgaria, Croatia, Cyprus, Malta, Romania, and Russia were excluded because yearly data for at least one macro-variable were missing.

### Micro-level Data and Variables

We selected those who were interviewed for at least two waves during the years 2010–2019 to model how individuals’ unemployment and temporary employment in year *t* affected their probability of union formation in year *t + 1*, and how such micro-level effects were moderated by macro-level labour market regulations.

The dependent variable—union formation—was a time-varying binary event dummy (0 = no event; 1 = union formation). Following Kalmijn (2011) and Bolano and Vignoli (2021), we focused on the formation of coresidential partnership unions, which included both marriages and non-marital cohabitations. The union transition was operationalized as whether a person lived without a partner in year *t* but with a partner in year *t* + *1*. The dataset was organized like an event-history dataset. We selected individuals aged between 15 and 45 years who lived without a partner in their first interview. All person-years in the following waves were kept in the sample until the event occurred, attrition, or the date of panel exit. However, a standard discrete-time event-history analysis was not appropriate for the EU-SILC data because the dataset’s short observation period and the left truncation of union status were not feasible to reconstruct a complete union history. Alternatively, we used a probability model that adjusted for individual ages at the baseline to estimate the effects of independent variables on the instantaneous transition probability of union status (see Kalmijn (2011) for similar applications). To examine gender heterogeneities, we stratified the sample by gender. The final sample for females included 87,670 person-years from 40,468 females who lived without a partner during their first interview, among whom 3223 formed a coresidential union in the subsequent years. The male sample included 115,666 person-years from 45,957 males, among whom 3077 formed a coresidential union.

The key independent variable was one’s employment status in year *t*. We used respondents’ self-defined working status to identify dependent employees, self-employees, the unemployed, and inactive individuals (i.e. students, further training, disabled or unfit to work, in compulsory military service, or fulfilling domestic tasks and care responsibilities). Because we focused on the micro–macro interactions between employment situations and labour market regulations, we excluded observations that were economically inactive (30.3% for female and 20.6% for male samples). According to different contract types, dependent employment was further differentiated into permanent employment (employed by open-ended contracts) and temporary employment (employed by fixed-term contracts). Taken together, the employment status variable comprised four categories: (1) permanent employment, (2) temporary employment, (3) unemployment, and (4) self-employment, where permanent employment served as the reference group.

We controlled for a set of micro-level confounding variables. First, educational attainment was controlled for in three categories: low (ISCED 0–2), middle (ISCED 3–4), and high (ISCED 5–8). Second, we controlled for individual’s self-related health status in three categories: good or very good, fair, and bad or very bad. Third, we controlled for the number of children at home in a binary dummy variable (“no child” versus “one or more children”). Fourth, to control for the impact of household wealth on individual work and partnering behaviours (Kravdal, 1999), we included two subjective measures on household economic situations. The first variable reflected the household financial ability to make ends meet, which was recategorized into a binary variable (“fairly easy, easy, or very easy” versus “with some difficulty, difficult, or very difficult” to make ends meet.) The second was a binary variable measuring whether a household could cope with unexpected financial expense (“yes” versus “no”). We chose these two subjective variables because they offered good comparability for European research on household wealth and income (Whelan & Maître, 2010). Finally, we included age and its squared term in the model to account for the baseline trend of work–family life course (Moen, 2018). All control variables were measured in year *t* to align with the key independent variable.

### Macro-level Data and Variables

At the macro-level, our key variables were four indicators measuring the three dimensions of labour market regulations. First, the strictness of EPLs was captured by the EPLR and EPLT indicators from the OECD.Stat. The EPLR indicator measured the strictness of regulations of individual dismissals of regular workers. We used Version 3 of the EPLR indicator, which incorporated nine items on different aspects of dismissal procedures (e.g. advanced notification, severance pay) and ranged from 0 (not regulated) to 6 (highly regulated). The EPLT indicator, ranging from 0 (not regulated) to 6 (highly regulated), reflected the regulations on using temporary contracts. The EPLT indicator (Version 3) incorporated eight items that measured the valid case of using fixed-term contracts, the maximum time duration and the number of consecutive usages of fixed-term contracts, and the regulation on temporary agency works, etc. Second, we captured the prevalence of collective bargaining institutions using the CBC rates from the OECD.Stat. The indicator was a continuous variable measured in percentage points, ranging from 0 to 100. Third, we measured the generosity of unemployment benefits using the net income replacement rates from the OECD.Stat. To emphasize its function as a longer-term income safety net for single individuals, we calculated the income replacement rates at the 18th month of unemployment for single persons without children who earned an average wage before unemployment. Sensitivity analyses in Table S7 showed that switching the unemployment duration to 12 or 24 months did not substantively change the results. Following the default setting, social assistance benefits and housing benefits were included to calculate the net replacement rate.

In theory, labour market institutions influence not only individuals’ employment outcomes but also their family behaviours (Blossfeld et al., 2005; Esping-Andersen & Regini, 2000; Scherer, 2009). Therefore, the four labour market indicators were always included in our models as control variables. Moreover, a set of macro-level control variables were included in our multilevel models. First, because individuals’ work and family behaviours are procyclical (Ebell, 2011; Sobotka et al., 2011) and are affected by the macro-level economic uncertainty (de Lange et al., 2014), we controlled for a country’s GDP per capita, unemployment rates, and temporary employment rates. Second, we controlled for female labour force participation rates as proxies of labour market gender egalitarianism because previous research showed that men’s unemployment discouraged union formation especially in a less gender egalitarian context (Kalmijn, 2011). Third, we controlled for the degree of economic globalization because globalization is arguably a driving factor behind the rising economic uncertainties and changing work–family life courses (Blossfeld et al., 2005). The macro-indicators of GDP per capita, sex-specific unemployment rates, temporary employment rates, and female labour force participation rates came from the Eurostat; the economic globalization indicator came from the KOF Globalization Index database (Gygli et al., 2019). Table 1 provides the descriptive statistics of all micro- and macro-level variables. Country-specific descriptive statistics of micro-level variables are presented in Table S2.

**Table 1:** Descriptive statistics of variables

	Female sample			Male sample		
**Dependent variable**						
Union formation probability	3.68%			2.66%		
Union formation event	3223			3077		
Exposure (person-years)	87,670			115,666		
	Mean; %	SD	Range	Mean; %	SD	Range
**Independent variables**						
*Micro-variables*						
Employment status						
Permanent employment	50.0%			47.0%		
Temporary employment	22.6%			18.9%		
Unemployment	21.6%			24.2%		
Self-employment	5.8%			10.0%		
Age	29.6	7.2	[15, 45]	29.2	7.1	[15, 45]
Educational level						
Low (ISCED 0–2)	13.4%			22.1%		
Middle (ISCED 3–4)	46.2%			54.5%		
High (ISCED 5–8)	40.4%			23.4%		
Health status						
Good/Very good	87.2%			89.3%		
Fair	10.7%			8.8%		
Bad/Very bad	2.1%			1.9%		
Number of children						
No child	85.9%			99.9%		
One or more children	14.1%			0.1%		
Ability to make ends meet						
Fairly easy	34.6%			34.3%		
With some difficulty/ difficult	65.4%			65.8%		
Capacity to face unexpected expense						
Yes	55.1%			57.3%		
No	44.9%			42.7%		
*Macro-variables*						
EPLR	2.3	0.5	[1.2, 3.9]	2.3	0.5	[1.2, 3.9]
EPLT	2.4	0.7	[0.4, 3.8]	2.4	0.7	[0.4, 3.8]
CBC rate	60.5	32.6	[6.1, 100.0]	60.5	32.6	[6.1, 100.0]
UB replacement rate	38.2	21.4	[0.0, 75.0]	38.2	21.4	[0.0, 75.0]
GDP per capita	27.1	10.1	[15.8, 79.0]	27.1	10.1	[15.8, 79.0]
Unemployment rate (by sex)	11.9	7.1	[2.6, 31.7]	11.4	5.7	[1.8, 25.6]
Temporary employment rate (by sex)	15.2	6.9	[1.3, 28.0]	14.0	7.0	[1.7, 28.5]
Economic globalization	77.4	6.4	[66.0, 90.0]	77.4	6.4	[66.0, 90.0]
Female labour force participation rate	61.9	9.1	[42.9, 84.5]	61.9	9.1	[42.9, 84.5]

### Methods

Throughout the paper, we specified linear probability models (LPM) of union formation. Three-level multilevel models were applied to capture the hierarchical data structure. Our first model estimated the European average effects of temporary employment and unemployment on the probability of union formation. Individual’s probability of union formation *y* at time t + 1 in country *j* was written as:

1 $$P\left( {y_{{{\text{j}}\left( {{\text{t}} + 1} \right){\text{i}}}} \; = \;1{|}{\varvec{X}}_{{{\text{jti}}}} , {\varvec{Z}}_{{{\text{jt}}}} } \right)\; = \;\beta_{0} \; + \;{\varvec{\beta}}_{{\mathbf{t}}} \; + \;{\varvec{X}}_{{{\text{jti}}}} {\varvec{\beta}}_{1} \; + \;{\varvec{Z}}_{{{\text{jt}}}} {\varvec{\beta}}_{2} \; + \;v_{{0{\text{j}}}} \; + \;u_{{0{\text{jt}}}} \; + \;e_{{{\text{jti}}}}$$

where $\beta_{0}$ is the grand average of the union formation probability and ${\varvec{\beta}}_{{\mathbf{t}}}$ presents the period fixed effects. The effects of micro-level variables ${\varvec{X}}_{{{\text{jti}}}}$ were denoted by ${\varvec{\beta}}_{1}$. The effects of macro-level variables ${\varvec{Z}}_{{{\text{jt}}}}$ were denoted by ${\varvec{\beta}}_{2}$. Unobserved heterogeneities in the outcome probabilities at different levels were captured by three variance components: $v_{{0{\text{j}}}}$ (country level), $u_{{0{\text{jt}}}}$ (country-wave level), and $e_{{{\text{jti}}}}$ (individual level).

The parameters of interests were ${\varvec{\beta}}_{1}$ corresponding to specific employment situations. A common modelling strategy is applying a multilevel mixed-effect model with random intercepts on Eq. (1), treating the unobserved heterogeneities at all levels as random components that are not correlated with the observed variables in the model (Heisig & Schaeffer, 2019). This approach, however, is usually problematic in cross-national comparative research because unobserved differences among countries may affect dependent and independent variables simultaneously (e.g. cultural norms). Therefore, we applied a country fixed effects model for multilevel analyses (Fairbrother, 2014). Instead of treating the country-level variances $v_{{0{\text{j}}}}$ as random intercepts, we directly controlled for these variances using a set of country dummy variables $\mathop \sum \limits_{{{\text{j}} = 1}}^{{{\text{N}} - 1}} \gamma_{{0{\text{j}}}} c_{{\text{j}}}$. This model ruled out confounding problems resulted from time-constant unobserved heterogeneities between countries.

Our second model investigated how such effects were moderated by country-level variations in labour market regulations. Specifically, we introduced cross-level interactions between employment and labour market regulations and allowed the effects of employment variables to vary across countries. This model was:

2 $$\begin{gathered} P\left( {y_{{{\text{j}}\left( {{\text{t}} + 1} \right){\text{i}}}} \; = \;1{|}{\varvec{X}}_{{{\text{jti}}}} , \;{\varvec{Z}}_{{{\text{jt}}}} } \right)\; = \;\beta_{0} \; + \;{\varvec{\beta}}_{{\mathbf{t}}} \; + \;{\varvec{X}}_{{{\text{jti}}}} {\varvec{\beta}}_{1} \; + \;{\varvec{Z}}_{{{\text{jt}}}} {\varvec{\beta}}_{2} \; + \;\left( {{\varvec{X}}_{{{\text{jti}}}} {\varvec{Z}}_{{{\text{jt}}}} } \right){\varvec{\beta}}_{3} \hfill \\ \quad \quad \quad \quad \quad + \;v_{{0{\text{j}}}} \; + \;{\varvec{X}}_{{{\text{jti}}}} {\varvec{v}}_{{1{\text{j}}}} \; + \;u_{{{\text{jt}}}} \; + \;e_{{{\text{jti}}}} \hfill \\ \end{gathered}$$

where the parameters ${\varvec{\beta}}_{3}$ for the cross-level interactions ${\varvec{X}}_{{{\text{jti}}}} {\varvec{Z}}_{{{\text{jt}}}}$ reflected the moderating effects of specific labour market indicators on the micro-level effects of employment situations on union formation. The country-level unobserved effect heterogeneity of employment situations on union formation are represented by ${\varvec{v}}_{{1{\text{j}}}}$.

For estimation, we applied the country fixed effects and fixed slopes model (cFES) (Giesselmann & Schmidt-Catran, 2019), which utilized within-country “changes” in labour market regulations to estimate their moderating effects on the micro-level relationship between employment and union formation. The cFES model was:

3 $$\begin{gathered} P\left( {y_{{{\text{j}}\left( {{\text{t}} + 1} \right){\text{i}}}} \; = \;1{|}{\varvec{X}}_{{{\text{jti}}}} , {\varvec{Z}}_{{{\text{jt}}}} } \right)\; = \;\beta_{0} \; + \;{\varvec{\beta}}_{{\mathbf{t}}} \; + \;{\varvec{X}}_{{{\text{jti}}}} {\varvec{\beta}}_{1} \; + \;{\varvec{Z}}_{{{\text{jt}}}} {\varvec{\beta}}_{2} \; + \;\left( {{\varvec{X}}_{{{\text{jti}}}} {\varvec{Z}}_{{{\text{jt}}}} } \right){\varvec{\beta}}_{3} \hfill \\ \quad \quad \quad \quad \quad + \;\mathop \sum \limits_{{{\text{j}} = 1}}^{{{\text{N}} - 1}} \gamma_{{0{\text{j}}}} c_{{\text{j}}} \; + \;\mathop \sum \limits_{{{\text{j}} = 1}}^{{{\text{N}} - 1}} \gamma_{{1{\text{j}}}} \left( {c_{{\text{j}}} {\varvec{X}}_{{{\text{jti}}}} } \right)\; + \;u_{{{\text{jt}}}} \; + \;e_{{{\text{jti}}}} \hfill \\ \end{gathered}$$

This model controlled for both the time-constant country-level unobserved heterogeneity in union formation probability using $\mathop \sum \limits_{{{\text{j}} = 1}}^{{{\text{N}} - 1}} \gamma_{{0{\text{j}}}} c_{{\text{j}}}$ and the time-constant effect heterogeneity of employment statuses across countries using $\mathop \sum \limits_{{{\text{j}} = 1}}^{{{\text{N}} - 1}} \gamma_{{1{\text{j}}}} \left( {c_{{\text{j}}} {\varvec{X}}_{{{\text{jti}}}} } \right)$. Accordingly, the cFES model yielded superior estimations of the macro-level moderating effects ${\varvec{\beta}}_{3}$ than the mixed-effects model because the latter treated both country-level heterogeneities as random intercepts and random slopes, assuming not only time-varying but also time-constant country-level unobserved heterogeneities to be uncorrelated with the independent variables. This assumption was problematic because some time-constant country-level unobserved heterogeneities, such as cultural values or gender norms, could correlate with labour market regulations and influence the effects of individual employment status (Kalmijn, 2011). Using the cFES model, we ruled out this source of estimation bias.

However, the cFES model had an empirical limitation because its estimation required within-country “changes” in macro-variables over time. In our data, some countries had relatively stable labour market regulations between the years 2010 and 2019. As a result, some moderating effect estimates from our cFES models could be inflated and overrepresent specific countries. To check the robustness of cFES, we performed a set of supplementary analyses and discussed their implications (more details in Appendix B).

## Results

### Descriptive Results

Table 2 provides country-specific descriptive statistics of key variables. First, we observed great institutional variations in labour market regulations between European countries. The nonzero standard deviations indicated that most countries had experienced labour market changes during the years 2010–2018. As illustrated in Fig. S1, the directions and sizes of labour market changes in each policy dimension differed significantly across countries. Taking EPLR for example, 13 out of the 26 countries had experienced EPLR changes, with the most significant deregulation observed in Portugal during the years 2012–2014. Changes in CBC and unemployment benefits were common as well, despite their smaller scales.

**Table 2:** Descriptive statistics of labour market indicators and union formation probabilities by country, average over years

Country	ISO Code	EPLR		EPLT		CBC rate		Unemployment benefits income replacement rate		Union formation probability by employment status			N (number of country-round)	n (number of obs.)
		Mean	SD	Mean	SD	Mean	SD	Mean	SD	Permanent workers (%)	Temporary workers (%)	Unemployed (%)		
Austria	AT	2.1	0.0	2.2	0.0	98.0	0.0	52.1	2.1	4.42	4.82	3.73	9	7087
Belgium	BE	2.1	0.2	2.2	0.1	96.0	0.0	51.4	0.8	6.71	6.73	3.85	9	6106
Switzerland	CH	1.3	0.0	1.5	0.0	48.0	5.3	71.0	0.0	6.09	2.37	3.63	7	3922
Czechia	CZ	3.2	0.1	2.1	0.1	35.2	1.4	30.7	0.6	2.49	1.68	1.81	9	5986
Germany	DE	2.4	0.0	1.8	0.1	55.2	1.0	34.6	0.8	4.36	1.84	2.38	4	2701
Denmark	DK	1.4	0.0	1.9	0.1	81.9	1.8	62.3	0.4	10.53	7.80	8.94	9	1143
Estonia	EE	1.7	0.0	2.8	0.4	17.5	5.6	24.3	1.2	4.53	2.37	3.40	9	4880
Spain	ES	2.0	0.1	3.2	0.1	80.9	1.9	56.8	1.4	3.94	3.18	1.86	9	20,576
Finland	FI	2.3	0.0	1.8	0.0	88.2	3.6	56.0	1.9	9.68	11.13	5.02	9	4612
France	FR	2.7	0.0	3.2	0.1	98.0	0.1	67.2	0.9	6.73	6.14	4.13	9	4594
Greece	GR	2.4	0.1	2.9	0.1	29.8	25.5	11.8	6.8	1.50	0.93	0.68	9	19,332
Hungary	HU	1.6	0.2	2.0	0.0	26.2	1.7	15.9	2.9	1.59	1.92	1.50	9	13,146
Ireland	IE	1.3	0.1	1.1	0.2	34.6	2.3	45.7	1.2	1.52	0.27	0.99	9	4736
Iceland	IS	1.9	0.0	1.4	0.1	90.0	0.0	46.0	2.6	7.27	6.07	6.40	7	1215
Italy	IT	2.7	0.2	2.6	0.2	100.0	0.0	17.5	21.1	2.26	1.03	0.89	9	29,841
Lithuania	LT	2.3	0.1	3.1	0.4	8.4	0.3	23.1	5.6	0.84	1.25	1.36	5	1539
Luxembourg	LU	2.2	0.0	3.8	0.0	57.1	1.2	45.7	0.6	3.31	1.89	1.41	8	4186
Latvia	LV	2.8	0.0	1.8	0.0	19.3	6.7	35.6	4.5	2.53	2.19	0.81	7	6031
Netherlands	NL	3.4	0.1	1.3	0.1	83.1	4.4	64.6	9.4	4.43	4.51	2.74	9	5654
Norway	NO	2.2	0.0	3.2	0.2	70.9	1.3	64.8	0.5	9.13	8.14	3.44	9	3500
Poland	PL	2.3	0.0	2.2	0.0	16.9	1.3	21.7	0.5	2.81	2.21	1.51	9	16,289
Portugal	PT	3.2	0.3	2.4	0.1	74.7	1.6	69.9	3.1	3.73	3.56	2.08	9	12,938
Sweden	SE	2.5	0.0	1.5	0.2	88.5	0.3	44.6	1.8	10.15	7.00	5.81	9	2796
Slovenia	SI	2.2	0.2	2.3	0.2	70.3	3.8	34.0	1.2	5.23	5.79	3.16	9	6171
Slovakia	SK	2.5	0.2	2.4	0.1	31.6	4.3	18.0	0.8	2.21	2.26	0.86	7	8314
UK	UK	1.5	0.1	0.5	0.1	29.0	1.5	37.8	0.9	3.70	1.81	1.57	8	6041
*All*		*2.3*	*0.5*	*2.4*	*0.7*	*60.5*	*32.6*	*38.2*	*21.4*	*3.81*	*3.01*	*2.85*	*215*	*203,336*

Table 2 also shows that individuals’ union formation probabilities differed by their employment situations. Pooling all countries, permanent workers had a higher union transition probability (3.81%) than temporary workers (3.01%) and the unemployed (2.85%). However, country variations were noticeable. Some countries (e.g. Sweden) had larger gaps in union formation probabilities between employment groups, while other countries (e.g. Ireland) had mild employment group differentials in union transition.

Table S3 presents the correlations between countries’ average levels of labour market regulations and mean union formation probabilities. In general, country levels of EPLR and EPLT were negligibly correlated with mean union formation probabilities of all employment groups, but CBC and unemployment benefits were positively, though weakly, correlated with mean union formation probabilities of all employment groups (Table S3, columns 1–3). Moreover, the correlations between country averages of labour market regulations and the gaps in union transition between employment groups were mostly negligible (Table S3, columns 4–5), except that CBC and unemployment benefits were negatively, though weakly, correlated with the union transition gaps between the unemployed and permanent workers for men. At first glance, these results contradicted our theoretical expectations because countries with higher levels of EPLs and CBC were not linked to more negative union transition gaps between non-permanent and permanent employment groups, and the negative gaps in union transition for men were even larger in countries with high levels of unemployment benefits. However, we should not conclude too early about the absence of labour market moderating effects because these results only reflected the static correlations between union transition gaps and labour market regulations by country means, in which confounding factors such as micro-level demographic characteristics and country-level heterogeneities were ignored. To investigate whether the union transition gaps between employment groups were influenced by changes in labour market regulations during the past decade, multilevel regressions proposed in the method section were necessary.

### Micro-level Effect of Employment Status on Union Formation

Table 3 presents the modelling results of union formation from country fixed effects models. On the European average, unstable employment situations had substantively negative and statistically significant effects on union formation for both sexes (supported H1). Compared to permanent employment, temporary employment and unemployment decreased women’s union formation probability by 0.6 and 1.1 percentage points respectively. Considering that the union formation probability of female permanent employees was 4.1 percentage points, such effects amounted to reductions in union formation probability by 14.6% (0.6/4.1) and 27% (1.1/4.1). The negative effects were even stronger for men (supported H2). Compared to permanently employed men, whose average union transition probability was 3.1% points, being temporarily employed and being unemployed decreased men’s union formation probability by 0.6 and 1.4 percentage points; such effects amounted to a 19.3% (0.6/3.1) and a 45.1% (1.4/3.1) reduction in the union transition probability.

**Table 3:** Country fixed effects linear probability models on union formation

	Females			Males		
	*β*	S.E	Pr(Y)	*β*	S.E	Pr(Y)
Employment status						
Permanent employment	Ref.		0.041	Ref.		0.031
Temporary employment	−0.006*	(0.002)	0.035	−0.006*	(0.002)	0.025
Unemployment	−0.011*	(0.002)	0.030	−0.014*	(0.002)	0.017
Self-employment	−0.002	(0.004)	0.038	0.002	(0.002)	0.033
Educational level						
Low (ISCED 0–2)	Ref.			Ref.		
Middle (ISCED 3–4)	−0.003	(0.002)		0.002	(0.002)	
High (ISCED 5–8)	0.004	(0.003)		0.008*	(0.003)	
Health status						
Good	Ref.			Ref.		
Fair	−0.004	(0.003)		−0.006*	(0.002)	
Bad	−0.004	(0.003)		−0.010*	(0.002)	
Number of children						
No child	Ref.			Ref.		
One or more children	0.012*	(0.003)		0.299*	(0.091)	
Ability to make ends meet						
Fairly easy	Ref			Ref.		
With some difficulty/ difficult	−0.005*	(0.002)		−0.003 +	(0.002)	
Capacity to cover unexpected expense						
No	Ref					
Yes	0.003	(0.002)		0.003 +	(0.002)	
Age	0.011*	(0.002)		0.010*	(0.002)	
Age2	−0.000*	(0.000)		−0.000*	(0.000)	
GDP per capita	−0.007	(0.004)		−0.007	(0.006)	
Unemployment rate (by sex)	−0.014*	(0.007)		−0.016*	(0.003)	
Temporary employment rate (by sex)	−0.003	(0.006)		−0.006	(0.005)	
Economic globalization	−0.012 +	(0.006)		−0.008	(0.005)	
Female labour force participation	−0.011	(0.010)		−0.012	(0.008)	
EPLR	−0.003	(0.003)		−0.004	(0.003)	
EPLT	−0.009	(0.007)		−0.007	(0.006)	
CBC	−0.002	(0.002)		−0.008*	(0.003)	
UB generosity	−0.005*	(0.002)		0.000	(0.002)	
Country fixed effects	✓			✓		
Period fixed effects	✓			✓		
Number of countries	87,670			115,666		
Number of country-waves	215			215		
Number of observations	26			26		
R-squared	0.0218			0.0236		

All models control for country and period fixed effects using the LSDV approach. Standard errors are clustered at the country level. Significance levels: + *p* < 0.1, * *p* < 0.05

In a supplementary analysis (see Fig. S2), we stratified the samples by educational levels (Low, Middle, and High) to examine the possible educational gradient. Previous studies have hypothesized that the negative effects of unstable employment on family formation could pertain to the higher educated, especially women, because they are generally more career-oriented and face higher opportunity costs of starting a family compared to the lower educated (de Lange et al., 2014; Kreyenfeld, 2010). Results in Fig. S2 largely supported this expectation. Compared to lower-educated women, the negative effects of temporary employment and unemployment on union formation were stronger for middle and highly educated women. For men, temporary employment depressed union formation to a similar magnitude across education groups. Yet, the strongest negative effect of unemployment on union formation was found among highly educated men. In summary, these findings were consistent with the major findings in Table 3: while the effects of unstable employment on union formation differed by education, their directions were mostly negative.

Table S4 presents another robustness analysis using multinomial logistic regression models, where we distinguished between the competing risks of non-marital cohabitation and marriage to check whether the negative effects of unstable employment applied to both channels of union formation. Results showed that temporarily employed and unemployed single women were less likely to transition to either cohabitation or marriage compared to permanently employed women. For single men, those who were temporarily employed or unemployed were less likely to form either cohabiting or marriage unions. These results justified our analytical design in Table 3’s models, indicating that unstable employment negatively affected women’s and men’s union formation regardless of the type of unions being considered.

### Moderating Roles of Labour Market Regulations

**Fig. 2:**
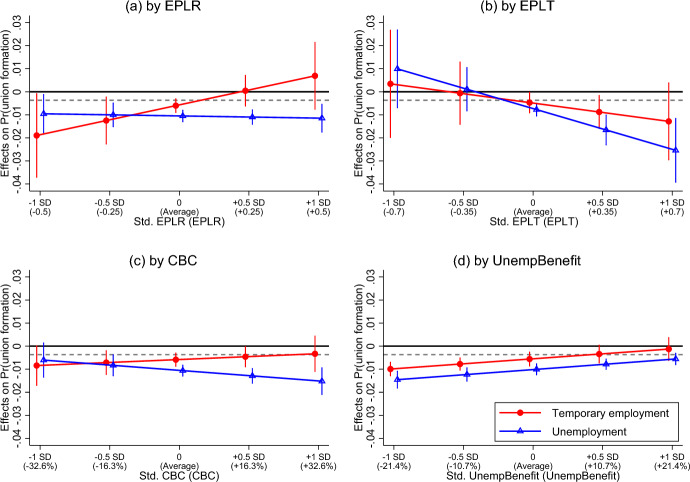
Effects of women’s temporary employment and unemployment on union formation by labour market regulations. Note: Point estimates with 95% confidence intervals. The major scale in the x-axis presents the standardized regulation indicators, with the corresponding original values presented in parentheses. The dashed line in each graph denotes a 10% decrease in union transition probability, which benchmarks the minimum value of substantive significance. See Table S5, Models 2–5 for the full estimation results

**Fig. 3:**
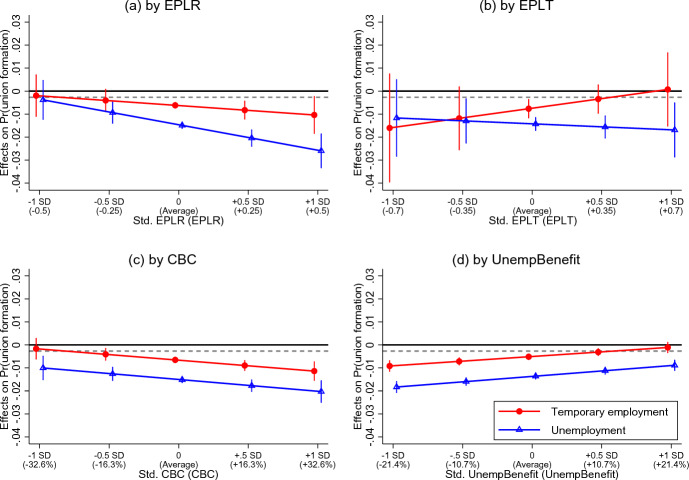
Effects of men’s temporary employment and unemployment on union formation by labour market regulations. Note: Point estimates with 95% confidence intervals. The major scale in the x-axis presents the standardized regulation indicators, with the corresponding original values presented in parentheses. The dashed line in each graph denotes a 10% decrease in union transition probability, which benchmarks the minimum value of substantive significance. See Table S6, Models 2–5 for the full estimation results

In this section, we further examined whether and how such a negative relationship between unstable employment and union formation was moderated by variations in labour market regulations. Tables S4 and S5 present full results of the cFES models for women and men. The coefficients of the interaction terms were standardized. For illustration, we plotted the estimated effects of both employment statuses (*y*-axis) against the standardized indicators of labour market regulations (*x*-axis) in Fig. 2 (for women) and Fig. 3 (for men). To evaluate the social significance of estimated effects, we plotted the benchmark values to compare against our point estimates and 95% confidence intervals across different policy scenarios. We defined a 10% decrease in the sample means of union formation probability as the minimum relevant values of substantive significance ($\theta_{{{\text{female}}}}$ = −0.00368; $\theta_{{{\text{male}}}}$ = −0.00266).

For women, Fig. 2a shows that the average negative effect of temporary employment on union formation became nearly zero with an increase of EPLR by 0.5 SD (+ 0.25); the effect even turned positive if EPLR increased by 1 SD (+ 0.5). On the other hand, the negative effects of women’s unemployment stayed at similar levels across different EPLR scenarios. In general, H3a and H3b were not supported by these results, although the wide 95% CIs indicated high statistical uncertainty. Figure 2b shows that stricter EPLT could reinforce the negative effects of women’s temporary employment and unemployment on union formation (supported H4a). Such a reinforcement effect was particularly strong in the negative relationship between women’s unemployment and union formation (supported H4b), while the reinforcement effect in the relationship between temporary employment and union formation was estimated with high uncertainty. Figure 2c indicates that the effect of women’s temporary employment on union formation was rather insensitive to changes in CBC, but higher CBC could reinforce the negative effect of women’s unemployment to some extent. In this regard, the negative reinforcement effect of higher CBC in H5a was only found in the relationship between women’s unemployment and union formation, which was in line with H5b’s prediction. Finally, Fig. 2d reveals that more generous unemployment benefits could attenuate the negative effect of women’s temporary employment or unemployment on union formation (supported H6). Specifically, one SD increase (+ 10.4%) in benefit replacement rates attenuated the average negative effects of women’s temporary employment and unemployment by 0.4 percentage point, which was substantively relevant for women’s union formation.

For men, Fig. 3a shows that stricter EPLR could reinforce the negative effects of men’s unstable employment situations on union formation (supported H3a), and such reinforcement effect was particularly strong in the negative relationship between unemployment and union formation (supported H3b). The negative effect of men’s unemployment on union formation probability strengthened from −0.015 to −0.026 when EPLR increased by one SD (+ 0.5). At odds with H4a, Fig. 3b shows that the negative effect of men’s temporary employment on union formation became insubstantial with the increase of EPLT by one SD (+ 0.7), although the wide 95% CIs indicated high uncertainty in such estimates. The moderating effect of stricter EPLT on the relationship between men’s unemployment and union formation was insignificant. Figure 3c shows that increases in CBC could substantively reinforce the negative effects of men’s unstable employment situations on union transition (supported H5a); such reinforcement operated on the negative effects of temporary employment and unemployment to a similar extent (contradicted H5b). In contrast, Fig. 3d shows that increasing generosity of unemployment benefits could substantively attenuate the negative effects of men’s temporary employment and unemployment on union formation (supported H6).

Finally, we observed gender nuances between the results in Figs. 2 and 3. As expected, stricter EPLR reinforced the negative effect of unstable employment on union formation only for men (supported H7), while stricter EPLT reinforced the negative effect only for women (supported H8).

With a theoretical focus on social inequalities in union formation, our analyses have so far centred around the “gaps” in union formation between permanent and non-permanent employment groups. The results generally supported the theoretical perspective that higher labour market flexibility (i.e. lower EPLR, lower EPLT, or fewer CBC) and more generous unemployment benefits may close the union formation gaps between employment groups. However, the gap-closing effects of labour market reforms could result from either an upward equalization (i.e. unstable employment individuals benefited more than permanent workers) or a downward equalization (i.e. permanent workers became worse off). To evaluate the holistic social influences of labour market reforms, Supplementary Fig. S3 (for women) and Fig. S4 (for men) present the predictive union formation probabilities by employment situations across different policy scenarios. In general, the gap-closing effects of higher labour market flexibility mainly reflected upward equalization, in which union formation probabilities increased for both permanent and non-permanent employment groups. On the other hand, the gap-closing effects of more generous unemployment benefits mainly reflected downward equalization, where permanent workers’ union formation probability had significantly reduced.

Finally, we performed a set of supplementary analyses to examine the robustness of our main results. Appendix A showed that our estimation on the moderating effects were generally not influenced by influential cases (outliers) where extreme labour market changes took place. The only exception was in the CBC analysis, where models using reduced samples (i.e. excluding Greece) produced very wide CIs, indicating high statistical uncertainty. Appendix B compared our cFES models with the mixed-effects models, showing that these two models yielded similar estimates of the labour market moderation effects. Appendix C selected a subset of countries that experienced substantial reforms in specific labour market regulations and modelled the time dynamics of the effects of unstable employment situations on union formation over time. In general, these case studies showed significant changes in the relationship between unstable employment and union formation following labour market changes, which provided additional supports to our main findings.

## Conclusion and Discussion

This study investigated the influences of individuals’ unstable employment situations, including temporary employment and unemployment, on the likelihood of union formation. It enriched the literature by highlighting the roles of labour market institutions in shaping the micro-level relationship between employment situations and partnership behaviours. Drawing on comparative panel data across 26 European countries and fixed effects multilevel models, our findings provided crucial implications for work–family theories and labour market policy research. Table S8 provides an overview of empirical supports for our hypotheses.

On the European average, we found that temporary employment and unemployment compared to permanent employment reduced people’s probability of forming a partnership union. This finding was in line with most studies for specific European countries (Bolano & Vignoli, 2021; de Lange et al., 2014; Landaud, 2021; Müller & Dräger, 2019; Oppenheimer, 2003; van Wijk et al., 2021; Vignoli et al., 2016), indicating that individuals in Europe tended to postpone union and family formation under the situations of employment uncertainty (Blossfeld et al., 2005; Kreyenfeld et al., 2012; Oppenheimer, 1988). Although the negative relationship was found for both genders, men’s union transition probabilities were reduced more than women’s by being temporarily employed (men’s 19.3% vs. women’s 14.6% reduction in transition risks) or unemployed (men’s 45.1% vs. women’s 27% reduction in transition risks). Similar to the finding of a gender-specific relationship between employment instability and fertility (Alderotti et al., 2021), the relationship between employment instability and union formation was gendered to some extent, despite the converging trend of work–family trajectories in Europe (Lesnard et al., 2016).

Breaking down the country-level development of such relationship, our analyses showed that the influences of temporary employment and unemployment on union formation may change according to the ongoing labour market reforms in many European countries. In general, our empirical results were in line with previous findings that stronger labour market insider–outsider segregation and weaker social welfare provisions could trigger more negative effects of unstable employment on union formation (Barbieri et al., 2015; Blossfeld et al., 2005; Scherer, 2009). First, we found that stricter employment protection legislations could reinforce the negative effects of unstable employment situations, especially unemployment, on union formation. Moreover, the reinforcement effect may operate through gender-specific channels. For men, higher EPLR reinforced the negative effects of unemployment on union formation. For women, the negative effect of unemployment on union formation was reinforced by stricter EPLT. Such a gender nuance of EPL’s moderating effects through different channels implied differentials in social expectations behind gender economic roles. Because men are socially expected to have a mature career before starting a family (Oppenheimer, 2003), the lowered possibility of transitioning to or being hired by a permanent contract due to stricter EPLR is particularly harmful for temporarily employed or unemployed men. In contrast, it is rather “socially acceptable” for women to be employed in temporary job when starting a family because they are more often expected to be secondary earners in a dual earning household (Van Lancker, 2012). As a result, when EPLT becomes stricter, higher difficulties of temporary contract renewals and fewer chances of transition out of unemployment via temporary jobs might be more discouraging for temporarily employed or unemployed women.

Second, higher CBC could reinforce the negative effects of men’s temporary employment and unemployment as well as women’s unemployment on union formation. The enlarged union formation gap echoed a recent finding about the role of CBC in escalating labour market insider–outsider dualism (Barton et al., 2021). However, we should not overinterpret the results because a robustness analysis showed that the estimated moderating effect of CBC in our multilevel models was substantially influenced by the CBC reform in Greece. Nonetheless, the patterns of non-positive moderating effects clearly deviated from the notion of an “inequality reducing” property of collective bargaining institutions (Hayter & Weinberg, 2011). To provide holistic policy suggestions, more studies are needed to evaluate collective bargaining’s social influences beyond wage distributions, such as the influence on people’s family lives.

Third, we found clear evidence that higher generosity of unemployment benefits may buffer the negative effects of temporary employment and unemployment on union formation for both genders. Previous sociological research has emphasized that generous unemployment benefits could reduce long-term income and employment disadvantages resulted from unstable employment experiences (Esping-Andersen & Regini, 2000; Gangl, 2004, 2006). We extended this literature beyond the discussion of economic well-being and showed that unemployment-related welfare provisions could level out the employment-driven social inequalities in demographic behaviours (Scherer, 2009), including union formation.

In the aftermath of the 2008/2009 Great Recession, the European Commission has been promoting labour market reforms under the notion of “flexicurity”, aiming to create higher employment flexibility and social security in a unified policy package (Eichhorst et al., 2017; Viebrock & Clasen, 2009). Policy suggestions that once prevailed in Europe in the 1990s such as deregulating EPLs, lowering CBC, and increasing unemployment benefits regained popularity. However, very few studies have discussed how these policies influence people’s social and family lives. To this end, our evaluations on the impacts of “flexicurity” reforms on employment–partnering relations bear crucial policy implications. The positive message was that flexibilization policies (i.e. deregulating EPLR/EPLT and lowering CBC), in general, could level out inequalities in union formation (Figs. 2 and 3) without sacrificing union transition probabilities for both the permanent and non-permanent employment groups (Figs. S3 and S4). On the other hand, while social security policies (i.e. more unemployment benefits) attenuated union formation inequalities across employment groups, such attenuation came from a scenario of downward equalization, where permanent workers’ union transition probability significantly declined. These findings suggested that “flexicurity” reforms should focus more on eliminating labour market segregation in strict employment systems and avoid overreliance on unemployment benefit provisions.

Before concluding this paper, we want to point out three unresolved issues and discuss possible directions for future research. First, while our country fixed effects multilevel models accounted for all country-level time-constant unobserved heterogeneities, the estimation results could still suffer from omitted variable biases due to unobserved heterogeneities at the individual level, such as one’s work–family preferences (Hakim, 2000). Ideally, a two-step multilevel estimation procedure that combines a central-demeaned individual fixed effects model at the first level and a country fixed effects model at the higher levels can solve the problem. However, we refrained from applying such a method to the EU-SILC dataset due to its short panel observation and insufficient outcome event transitions. Alternatively, we specified a discrete-time linear probability model at the micro-level to estimate the short-term effects of temporary employment and unemployment on the probability of union formation in the following year. Relatedly, whether the short-term effects found in our analyses apply to the long-term relationship between employment trajectories and cumulative union formation probabilities remains an open question. Future research may consider using other panel datasets that cover longer partnership histories. Nevertheless, due to its large scope and prospective panel design, the EU-SILC panel is still one of the most ideal datasets for comparative life course research in Europe.

Second, we might over-simplify the measurements of labour market regulations at the country level. Some researchers have pointed out the importance of considering the multidimensionality of labour market policy measures. For example, rather than using the original EPLR and EPLT indexes provided by OECD, which are weighted indexes comprising multiple items, Balz (2017) suggests using specific items of the indexes to capture the strictness of regulation in specific aspects (e.g. the definition of unfair dismissal). Regarding CBC measurements, research on industrial relations has shown that it is not only the CBC rate, but also the level of collective bargaining (Hayter & Weinberg, 2011) and the degree of bargaining centralization (Barton et al., 2021) that determine the degree of wage and employment coordination. As for measuring the comprehensiveness of unemployment benefits, the entitlement, coverage, and duration are also important aspects besides the generosity measured by the income replacement rates (Gangl, 2006). In summary, while our study has covered the most common measurements of labour market regulations in these three dimensions (Esping-Andersen & Regini, 2000), there is still room for improvement by considering the multidimensionality of specific policy measures.

Third, the current study has not yet discussed the complex influences of policy constellations on people’s work–family behaviours (Korpi et al., 2013). Our models were designed to quantify the moderating effects of each specific labour market regulation by holding other regulation indicators constant. However, different labour market institutions might complement each other in shaping the scale of social inequalities between labour market insiders and outsiders. For example, Barbieri and Cutuli (2016) and Högberg et al. (2019) found that it is the dualistic development of employment protection schemes, measured as the gap between EPLR and EPLT, that marginalizes the labour market outsiders and enlarges the insecurity gaps. Similarly, strict EPL combined with a strong collective bargaining institution might enhance labour market segregation and reinforce employment and income inequalities across employment status groups. Finally, unemployment benefits can more effectively buffer the unemployment wage scars in “a relatively flexible labour market that provides lower levels of employment protection” (Gangl, 2006: 1003), which might lead to an even weaker effect of unemployment on union formation.

In conclusion, our fruitful findings and the unresolved issues call for greater attention to the institutional influences of labour market regulations in the changing work–family dynamics in Europe. Building on and going beyond previous comparative research (Barbieri et al., 2015; Blossfeld et al., 2005; Karabchuk, 2020; Scherer, 2009), this study demonstrated that the societal-level transitions of the work–family relationship in Europe could be, at least partially, shaped by changes in labour market regulations in the past decade.

## Supplementary Information

Below is the link to the electronic supplementary material.

Supplementary file1 (DOCX 283 kb)
