# Supplementary material for: A Precarious Path to Partnership? The Moderating Effects of Labour Market Regulations on the Relationship Between Unstable Employment and Union Formation in Europe
Source: Eur J Popul. 2024 Mar 29;40(1):12. doi: 10.1007/s10680-024-09698-3 (PMC10980669; doi:10.1007/s10680-024-09698-3)
Supplement: Supplementary file 1 — Supplementary file1 (DOCX 283 kb) [file 10680_2024_9698_MOESM1_ESM.docx]

**A precarious path to partnership? The moderating effects of labour market regulations on the relationship between unstable employment and union formation in Europe**

**[Supplementary Materials]**

**Table S1** Theoretical mechanisms and hypotheses of the policy moderating effects

**Table S2** Descriptive analysis of micro variables by country, average over years

**Table S3** Correlations between countries’ average levels of labour market regulations and mean union formation probabilities

**Table S4** Results from the country fixed effects multinomial logit models, relative risk ratio

**Table S5** Results from country FES linear probability models, full table for women

**Table S6** Results from country FES linear probability models, full table for men

**Table S7** Sensitivity analysis: Results comparison of unemployment benefits’ moderating effects across different unemployment duration.

**Table S8** Overview of hypotheses and empirical supports

**Fig S1** Changes in labour market regulations across country

**Fig S2** Effects of temporary employment and unemployment on union formation probability by educational levels

**Fig S3** Predictive probability of union formation for women by employment status

**Fig S4** Predictive probability of union formation for men by employment status

**Appendix A** Influential cases (outliers) analysis

**Appendix B** Estimation results comparison between cFES and mixed-effects models

**Appendix C** Case studies of labour market moderating effects

**Table S1. Theoretical mechanisms and hypotheses of the policy moderating effects**

| **Labour market regulations ↑** | **Insecurity gap between temporary employment and permanent employment** | **Insecurity gap between unemployment and permanent employment** | **Hypotheses: moderating roles of labour market regulations** |
| --- | --- | --- | --- |
| **EPL for dismissing regular workers (EPLR)** | *Employment insecurity gap ↑*   - Higher costs of dismissal discourage the transition to permanent contract | *Employment insecurity gap ↑*   - Higher costs of dismissal discourage hiring - Stronger scar effects due to prolonged unemployment duration | *H3a: Stricter EPLR reinforces the negative effect of unstable employment on union formation.*  *H3b: The reinforcement effect of stricter EPLR is particularly strong in the negative relationship between unemployment and union formation.* |
| **EPL for using temporary contract (EPLT)** | *Employment insecurity gap* ↑ (↓)   - Shorter temporary contract duration and lower possibility of temporary contract renewal - More comprehensive temporary employee protection | *Employment insecurity gap ↑*   - Higher costs of hiring temporary employees discourage reemployment in temporary positions | *H4a: Stricter EPLT reinforces the negative effect of unstable employment on union formation.*  *H4b: The reinforcement effect of stricter EPLT is particularly strong in the negative relationship between unemployment and union formation.* |
| **Collective bargaining agreement coverage rate (CBC)** | *Income insecurity gap* (↓)   - Higher bargaining power over wages, benefits, and insurance   *Employment insecurity gap* ↑   - Enhanced insider-outsider dualism | *Employment insecurity gap ↑*   - Enhanced insider-outsider dualism | *H5a: Higher CBC reinforces the negative effect of unstable employment on union formation.*  *H5b: The reinforcement effect of higher CBC is particularly strong in the negative relationship between unemployment and union formation.* |
| **Generosity of unemployment benefits** | *Income insecurity gap ↓*   - Income safety net after the end of temporary contract   *Employment insecurity gap* ↓   - Increased subsequent job tenure and higher probability of permanent contract transition | *Income insecurity gap ↓*   - Income safety net   *Employment insecurity gap* ↓   - Weaker scar effects by permitting workers to search for adequate reemployment | *H6: More generous unemployment benefits attenuate the negative effect of unstable employment on union formation.* |

**Table S2. Descriptive analysis of micro variables by country, average over years**

| **Female sample** | Age (Mean) | Employment status | | | | Educational level | | | Health status | | | Have children | No capacity to cover unexpected expense | Have some difficulty to make ends meet |
| --- | --- | --- | --- | --- | --- | --- | --- | --- | --- | --- | --- | --- | --- | --- |
|  |  | Perm. | Temp. | Unemp. | Self-empl. | Low | Middle | High | Good | Fair | Bad |  |  |  |
| Austria | 29.8 | 74.0% | 11.3% | 10.7% | 4.0% | 9.8% | 57.0% | 33.2% | 86.7% | 11.1% | 2.2% | 16.5% | 28.5% | 40.2% |
| Belgium | 30.0 | 57.1% | 21.5% | 17.5% | 3.9% | 13.4% | 35.2% | 51.4% | 86.7% | 9.8% | 3.5% | 19.3% | 33.3% | 44.3% |
| Switzerland | 25.9 | 60.6% | 33.4% | 3.5% | 2.5% | 21.1% | 49.5% | 29.4% | 88.4% | 10.4% | 1.2% | 3.6% | 18.4% | 24.3% |
| Czechia | 29.7 | 63.3% | 19.6% | 11.1% | 6.0% | 6.7% | 62.8% | 30.4% | 90.9% | 7.5% | 1.5% | 20.4% | 43.7% | 65.5% |
| Germany | 30.4 | 58.5% | 29.7% | 9.1% | 2.7% | 12.5% | 54.2% | 33.3% | 83.6% | 13.6% | 2.9% | 14.6% | 44.4% | 17.0% |
| Denmark | 32.4 | 76.4% | 13.1% | 9.2% | 1.3% | 9.0% | 38.1% | 52.9% | 82.2% | 14.8% | 3.0% | 25.0% | 34.2% | 25.2% |
| Estonia | 30.0 | 79.2% | 7.0% | 11.1% | 2.7% | 12.1% | 47.2% | 40.7% | 81.9% | 15.2% | 2.9% | 32.7% | 49.8% | 64.9% |
| Spain | 29.9 | 31.5% | 30.2% | 33.5% | 4.9% | 28.8% | 23.2% | 48.0% | 90.8% | 8.0% | 1.2% | 10.1% | 42.9% | 64.1% |
| Finland | 29.9 | 54.3% | 26.7% | 12.0% | 7.0% | 7.2% | 49.9% | 42.9% | 83.3% | 15.2% | 1.4% | 17.0% | 46.7% | 29.5% |
| France | 30.0 | 50.5% | 26.9% | 19.6% | 3.0% | 11.3% | 47.4% | 41.4% | 79.9% | 16.5% | 3.6% | 22.7% | 46.4% | 67.9% |
| Greece | 29.1 | 26.9% | 20.4% | 44.1% | 8.6% | 4.7% | 46.9% | 48.4% | 96.1% | 3.0% | 0.9% | 0.9% | 51.3% | 93.0% |
| Hungary | 28.8 | 65.1% | 14.4% | 17.5% | 3.0% | 10.4% | 55.0% | 34.6% | 88.8% | 9.5% | 1.7% | 12.7% | 67.8% | 87.7% |
| Ireland | 29.5 | 60.1% | 18.4% | 18.8% | 2.7% | 8.5% | 41.0% | 50.5% | 93.0% | 6.1% | 0.9% | 26.5% | 58.1% | 68.0% |
| Iceland | 29.1 | 69.2% | 17.6% | 8.9% | 4.3% | 26.5% | 37.7% | 35.8% | 86.5% | 12.0% | 1.4% | 37.7% | 54.2% | 56.7% |
| Italy | 30.8 | 40.4% | 19.7% | 27.4% | 12.5% | 16.1% | 54.3% | 29.6% | 91.7% | 6.5% | 1.8% | 7.3% | 39.4% | 76.0% |
| Lithuania | 29.2 | 74.6% | 4.6% | 16.3% | 4.5% | 8.4% | 39.8% | 51.8% | 82.0% | 15.7% | 2.3% | 19.9% | 50.2% | 79.2% |
| Luxembourg | 29.1 | 63.6% | 20.9% | 11.9% | 3.6% | 24.3% | 39.9% | 35.8% | 81.8% | 14.2% | 4.0% | 15.8% | 30.1% | 34.3% |
| Latvia | 29.9 | 76.7% | 5.2% | 15.0% | 3.0% | 12.3% | 47.6% | 40.0% | 74.8% | 23.1% | 2.1% | 28.6% | 66.7% | 79.0% |
| Netherlands | 31.6 | 63.2% | 25.2% | 5.9% | 5.6% | 8.1% | 36.9% | 55.0% | 84.5% | 12.9% | 2.6% | 16.2% | 31.3% | 27.0% |
| Norway | 30.1 | 66.1% | 23.8% | 6.2% | 3.8% | 17.9% | 27.8% | 54.3% | 86.2% | 10.4% | 3.4% | 19.6% | 31.5% | 23.6% |
| Poland | 28.0 | 35.0% | 40.1% | 19.3% | 5.5% | 5.7% | 53.8% | 40.5% | 88.1% | 10.0% | 1.9% | 14.4% | 50.8% | 71.2% |
| Portugal | 29.3 | 39.2% | 33.0% | 23.1% | 4.7% | 25.6% | 37.8% | 36.6% | 74.5% | 22.6% | 2.9% | 15.3% | 45.4% | 78.0% |
| Sweden | 29.2 | 60.4% | 27.5% | 10.3% | 1.8% | 6.1% | 47.4% | 46.5% | 84.3% | 12.6% | 3.1% | 18.9% | 30.0% | 20.4% |
| Slovenia | 31.5 | 51.1% | 20.6% | 23.3% | 5.0% | 4.9% | 51.1% | 44.1% | 79.5% | 16.5% | 4.0% | 24.5% | 48.2% | 68.9% |
| Slovakia | 27.8 | 61.0% | 17.7% | 18.2% | 3.1% | 3.5% | 56.2% | 40.4% | 92.8% | 6.2% | 1.0% | 5.1% | 35.0% | 76.7% |
| UK | 27.8 | 74.0% | 11.2% | 10.1% | 4.7% | 9.8% | 52.6% | 37.5% | 84.2% | 12.8% | 3.0% | 26.8% | 52.0% | 57.3% |

**Table S2.** (*Continued*)

| **Male**  **sample** | Age (Mean) | Employment status | | | | Educational level | | | | Health status | | | Have children | No capacity to cover unexpected expense | Have some difficulty to make ends meet |
| --- | --- | --- | --- | --- | --- | --- | --- | --- | --- | --- | --- | --- | --- | --- | --- |
|  |  | Perm. | Temp. | Unemp. | Self-empl. | Low | | Middle | High | Good | Fair | Bad |  |  |  |
| Austria | 29.3 | 59.8% | 14.3% | 18.9% | 7.0% | | 18.4% | 46.0% | 35.6% | 91.9% | 6.4% | 1.7% | 0.3% | 28.4% | 42.7% |
| Belgium | 26.8 | 60.2% | 29.8% | 5.8% | 4.3% | | 22.5% | 52.1% | 25.4% | 90.7% | 8.4% | 0.9% | 0.0% | 16.6% | 24.1% |
| Switzerland | 30.0 | 66.2% | 13.4% | 11.1% | 9.4% | | 5.8% | 76.8% | 17.4% | 91.1% | 6.9% | 2.0% | 0.0% | 39.5% | 61.2% |
| Czechia | 28.6 | 56.3% | 31.1% | 9.8% | 2.9% | | 20.2% | 56.5% | 23.3% | 88.1% | 10.6% | 1.4% | 0.0% | 33.6% | 18.5% |
| Germany | 30.5 | 71.7% | 11.7% | 12.2% | 4.4% | | 20.1% | 47.7% | 32.2% | 82.2% | 14.1% | 3.6% | 0.3% | 25.5% | 22.9% |
| Denmark | 28.5 | 66.9% | 6.8% | 21.5% | 4.8% | | 25.9% | 58.1% | 16.0% | 81.0% | 16.2% | 2.8% | 0.0% | 44.0% | 64.6% |
| Estonia | 29.7 | 30.2% | 24.1% | 37.0% | 8.8% | | 44.5% | 24.2% | 31.3% | 93.0% | 6.0% | 1.1% | 0.1% | 43.4% | 66.7% |
| Spain | 30.1 | 51.0% | 15.3% | 20.6% | 13.2% | | 12.2% | 63.7% | 24.1% | 83.1% | 14.4% | 2.5% | 0.0% | 31.9% | 22.6% |
| Finland | 28.1 | 41.7% | 28.7% | 24.5% | 5.0% | | 18.3% | 50.9% | 30.8% | 84.5% | 13.3% | 2.2% | 0.3% | 39.3% | 63.0% |
| France | 30.7 | 25.2% | 17.9% | 38.6% | 18.3% | | 13.9% | 56.2% | 30.0% | 96.1% | 2.7% | 1.2% | 0.0% | 50.2% | 93.6% |
| Greece | 28.5 | 61.0% | 15.5% | 19.7% | 3.9% | | 13.7% | 70.3% | 16.0% | 89.8% | 8.3% | 2.0% | 0.0% | 73.1% | 88.6% |
| Hungary | 28.5 | 42.7% | 13.2% | 36.7% | 7.3% | | 19.2% | 44.3% | 36.5% | 93.4% | 5.8% | 0.7% | 0.1% | 54.5% | 70.2% |
| Ireland | 27.8 | 65.2% | 17.6% | 11.2% | 6.0% | | 41.1% | 43.0% | 15.8% | 85.4% | 13.1% | 1.5% | 0.0% | 30.2% | 36.6% |
| Iceland | 30.5 | 39.1% | 14.4% | 29.7% | 16.9% | | 27.3% | 56.1% | 16.6% | 92.2% | 5.8% | 2.0% | 0.0% | 38.2% | 74.9% |
| Italy | 28.3 | 65.7% | 5.7% | 21.5% | 7.1% | | 16.3% | 57.2% | 26.5% | 82.5% | 15.7% | 1.8% | 0.2% | 51.7% | 80.5% |
| Lithuania | 28.0 | 62.1% | 22.0% | 11.9% | 4.0% | | 28.8% | 48.2% | 22.9% | 86.1% | 11.0% | 2.8% | 0.3% | 24.6% | 29.2% |
| Luxembourg | 28.4 | 63.0% | 6.7% | 24.6% | 5.7% | | 26.7% | 56.1% | 17.2% | 78.8% | 18.6% | 2.6% | 0.1% | 66.9% | 81.0% |
| Latvia | 31.3 | 63.6% | 21.0% | 5.7% | 9.7% | | 15.7% | 41.9% | 42.4% | 90.8% | 8.3% | 0.9% | 0.0% | 19.8% | 18.6% |
| Netherlands | 29.0 | 67.0% | 18.6% | 8.4% | 6.1% | | 29.3% | 40.4% | 30.3% | 84.2% | 13.0% | 2.8% | 0.0% | 22.9% | 16.5% |
| Norway | 27.5 | 31.3% | 34.7% | 20.9% | 13.1% | | 12.9% | 69.7% | 17.3% | 89.5% | 8.4% | 2.2% | 0.0% | 51.6% | 75.0% |
| Poland | 28.9 | 35.9% | 29.0% | 29.7% | 5.3% | | 49.4% | 33.5% | 17.0% | 76.8% | 20.3% | 2.8% | 0.2% | 44.3% | 80.1% |
| Portugal | 28.9 | 63.8% | 19.7% | 12.7% | 3.8% | | 8.2% | 66.1% | 25.7% | 88.1% | 10.5% | 1.4% | 0.0% | 20.4% | 11.2% |
| Sweden | 30.4 | 52.6% | 18.1% | 21.3% | 8.1% | | 11.9% | 67.8% | 20.3% | 84.5% | 12.2% | 3.3% | 0.3% | 44.6% | 68.6% |
| Slovenia | 27.8 | 56.8% | 15.0% | 20.5% | 7.7% | | 4.8% | 73.4% | 21.8% | 93.1% | 5.6% | 1.3% | 0.0% | 35.2% | 76.9% |
| Slovakia | 27.3 | 69.2% | 9.2% | 14.5% | 7.1% | | 14.8% | 51.7% | 33.5% | 88.6% | 9.5% | 1.9% | 0.0% | 41.4% | 48.2% |
| UK | 29.2 | 47.0% | 18.9% | 24.2% | 10.0% | | 22.1% | 54.5% | 23.4% | 89.3% | 8.8% | 1.9% | 0.1% | 42.7% | 65.8% |

**Table S3. Correlations between countries’ average levels of labour market regulations and mean union formation probabilities**

|  | Female | | | | |
| --- | --- | --- | --- | --- | --- |
|  | (1)  Pr(Union) of permanent workers | (2)  Pr(Union) of  temporary workers | (3)  Pr(Union) of unemployed | (2) ─ (1)  Diff in Pr(Union) between temporary and permanent workers | (3) ─ (1)  Diff in Pr(Union) between unemployed and permanent workers |
| EPLR | -0.109 | 0.013 | -0.070 | 0.099 | -0.006 |
| EPLT | -0.037 | 0.006 | -0.005 | 0.035 | 0.022 |
| CBC rate | 0.414* | 0.367* | 0.205* | 0.092 | -0.040 |
| UB replacement rate | 0.424* | 0.337* | 0.238* | 0.049 | -0.007 |
|  | Male | | | | |
|  | (1)  Pr(Union) of permanent workers | (2)  Pr(Union) of  temporary workers | (3)  Pr(Union) of unemployed | (2) ─ (1)  Diff in Pr(Union) between temporary and permanent workers | (3) ─ (1)  Diff in Pr(Union) between unemployed and permanent workers |
| EPLR | -0.038 | 0.036 | -0.083 | 0.074 | -0.018 |
| EPLT | -0.094 | 0.045 | -0.156* | 0.141 | -0.024 |
| CBC rate | 0.414* | 0.322* | 0.224* | -0.045 | -0.237* |
| UB replacement rate | 0.388* | 0.277* | 0.206* | -0.068 | -0.223* |

*Note*: Significance levels: * p < 0.05

**Table S4. Results from the country fixed effects multinomial logit models, relative risk ratio**

|  | Women | |  | Men | |
| --- | --- | --- | --- | --- | --- |
|  | Cohabiting / Single | Married / Single |  | Cohabiting / Single | Married / Single |
| Employment status |  |  |  |  |  |
| (Ref.=Permanent empl.) |  |  |  |  |  |
| Temporary empl. | 0.892* | 0.760* |  | 0.803* | 0.803* |
|  | (0.052) | (0.101) |  | (0.078) | (0.078) |
| Unemployment | 0.686* | 0.662* |  | 0.489* | 0.413* |
|  | (0.067) | (0.046) |  | (0.102) | (0.089) |
| Self-employment | 0.923 | 0.990 |  | 1.082 | 1.080 |
|  | (0.102) | (0.162) |  | (0.070) | (0.119) |
| Educational level |  |  |  |  |  |
| (Ref.=Low (ISCED 0-2)) |  |  |  |  |  |
| Middle (ISCED 3-4) | 0.928 | 0.988 |  | 1.213+ | 0.992 |
|  | (0.099) | (0.087) |  | (0.101) | (0.112) |
| High (ISCED 5-8) | 1.109 | 1.243 |  | 1.526* | 1.098 |
|  | (0.108) | (0.140) |  | (0.107) | (0.202) |
| Health status (Ref.=Good) |  |  |  |  |  |
| Fair | 0.877 | 0.884 |  | 0.772* | 0.836+ |
|  | (0.084) | (0.112) |  | (0.090) | (0.104) |
| Bad | 0.994 | 0.786 |  | 0.656* | 0.433* |
|  | (0.103) | (0.279) |  | (0.165) | (0.251) |
| Number of children (Ref.= no child) |  |  |  |  |  |
| One or more children | 1.473* | 1.418* |  | 27.652* | 20.390* |
|  | (0.099) | (0.136) |  | (0.627) | (0.562) |
| Ability to make ends meet |  |  |  |  |  |
| (Ref.=Fairly easy) |  |  |  |  |  |
| With some difficulty/ difficult | 0.837* | 0.969 |  | 0.874+ | 0.979 |
|  | (0.059) | (0.093) |  | (0.071) | (0.091) |
| Capacity to face unexpected expense |  |  |  |  |  |
| Yes | 1.087 | 1.049 |  | 1.142* | 1.051 |
|  | (0.069) | (0.059) |  | (0.066) | (0.093) |
| Age | 1.435* | 1.479* |  | 1.472* | 1.794* |
|  | (0.047) | (0.053) |  | (0.048) | (0.052) |
| Age2 | 0.994* | 0.993* |  | 0.994* | 0.991* |
|  | (0.001) | (0.001) |  | (0.001) | (0.001) |
| Period fixed effects | ✓ | ✓ |  | ✓ | ✓ |
| Country fixed effects | ✓ | ✓ |  | ✓ | ✓ |
| Pseudo R^2^ | 0.088 |  |  | 0.101 |  |
| n (individuals) | 60,834 |  |  | 75,602 |  |
| N (country-rounds) | 215 |  |  | 215 |  |
| M (countries) | 26 |  |  | 26 |  |

*Note*: The coefficients were relative risk ratios of the occurrence of a specific event compared to staying single in time t+1. Standard errors were clustered at the country level. Significance levels: + p<0.1, * p < 0.05.

**Table S5. Results from country FES linear probability models, full table for women**

|  | Model 1 | |  | Model 2 | |  | Model 3 | |  | Model 4 | |  | Model 5 | |
| --- | --- | --- | --- | --- | --- | --- | --- | --- | --- | --- | --- | --- | --- | --- |
|  | *β* | *S.E.* |  | *β* | *S.E.* |  | *β* | *S.E.* |  | *β* | *S.E.* |  | *β* | *S.E.* |
| Employment status (Ref.=Permanent employment) |  |  |  |  |  |  |  |  |  |  |  |  |  |  |
| Temporary employment | -0.006* | (0.001) |  | -0.006* | (0.001) |  | -0.005* | (0.002) |  | -0.006* | (0.001) |  | -0.005* | (0.001) |
| Unemployment | -0.010* | (0.001) |  | -0.010* | (0.001) |  | -0.008* | (0.001) |  | -0.011* | (0.001) |  | -0.010* | (0.001) |
| self-employment | 0.000 | (0.000) |  | 0.000 | (0.000) |  | 0.000 | (0.000) |  | 0.000 | (0.000) |  | 0.000 | (0.000) |
| Micro-level control variables | ✓ |  |  | ✓ |  |  | ✓ |  |  | ✓ |  |  | ✓ |  |
| Period fixed effects | ✓ |  |  | ✓ |  |  | ✓ |  |  | ✓ |  |  | ✓ |  |
| Country fixed effects | ✓ |  |  | ✓ |  |  | ✓ |  |  | ✓ |  |  | ✓ |  |
| Country fixed slopes | ✓ |  |  | ✓ |  |  | ✓ |  |  | ✓ |  |  | ✓ |  |
| Macro-level variables |  |  |  |  |  |  |  |  |  |  |  |  |  |  |
| GDP per capita | -0.007 | (0.004) |  | -0.007 | (0.004) |  | -0.008 | (0.004) |  | -0.007 | (0.004) |  | -0.007 | (0.004) |
| Unemployment rate (female) | -0.014* | (0.007) |  | -0.014* | (0.007) |  | -0.014* | (0.007) |  | -0.014* | (0.007) |  | -0.014* | (0.007) |
| Temporary employment rate (female) | -0.003 | (0.006) |  | -0.003 | (0.006) |  | -0.003 | (0.006) |  | -0.003 | (0.006) |  | -0.003 | (0.006) |
| Economic globalization | -0.012+ | (0.006) |  | -0.011+ | (0.006) |  | -0.012+ | (0.006) |  | -0.012+ | (0.006) |  | -0.011+ | (0.006) |
| Female labour force participation rate | -0.011 | (0.010) |  | -0.012 | (0.009) |  | -0.011 | (0.010) |  | -0.011 | (0.010) |  | -0.012 | (0.010) |
| EPLR | -0.002 | (0.003) |  | -0.005 | (0.004) |  | -0.002 | (0.003) |  | -0.002 | (0.003) |  | -0.002 | (0.003) |
| EPLT | -0.008 | (0.007) |  | -0.008 | (0.007) |  | -0.004 | (0.008) |  | -0.008 | (0.007) |  | -0.008 | (0.007) |
| CBC | -0.003 | (0.002) |  | -0.003 | (0.002) |  | -0.003 | (0.002) |  | -0.002 | (0.003) |  | -0.003 | (0.002) |
| UB generosity | -0.005* | (0.002) |  | -0.005* | (0.002) |  | -0.005* | (0.002) |  | -0.005* | (0.002) |  | -0.008* | (0.002) |
| Temp. empl. * EPLR |  |  |  | 0.013 | (0.008) |  |  |  |  |  |  |  |  |  |
| Unempl. * EPLR |  |  |  | -0.001 | (0.003) |  |  |  |  |  |  |  |  |  |
| Temp. empl. * EPLT |  |  |  |  |  |  | -0.008 | (0.010) |  |  |  |  |  |  |
| Unempl. * EPLT |  |  |  |  |  |  | -0.018* | (0.007) |  |  |  |  |  |  |
| Temp. empl. * CBC |  |  |  |  |  |  |  |  |  | 0.003 | (0.004) |  |  |  |
| Unempl. * CBC |  |  |  |  |  |  |  |  |  | -0.005+ | (0.003) |  |  |  |
| Temp. empl. * UB generosity |  |  |  |  |  |  |  |  |  |  |  |  | 0.004* | (0.001) |
| Unempl. * UB generosity |  |  |  |  |  |  |  |  |  |  |  |  | 0.004* | (0.001) |
| n (individuals) | 87,670 |  |  | 87,670 |  |  | 87,670 |  |  | 87,670 |  |  | 87,670 |  |
| N (country-rounds) | 215 |  |  | 215 |  |  | 215 |  |  | 215 |  |  | 215 |  |
| M (countries) | 26 |  |  | 26 |  |  | 26 |  |  | 26 |  |  | 26 |  |

*Note*: Significance levels: + p<0.1, * p < 0.05. Standard errors were clustered at the country level.

**Table S6. Results from country FES linear probability models, full table for men**

|  | Model 1 | |  | Model 2 | |  | Model 3 | |  | Model 4 | |  | Model 5 | |
| --- | --- | --- | --- | --- | --- | --- | --- | --- | --- | --- | --- | --- | --- | --- |
|  | *β* | *S.E.* |  | *β* | *S.E.* |  | *β* | *S.E.* |  | *β* | *S.E.* |  | *β* | *S.E.* |
| Employment status (Ref.=Permanent employment) |  |  |  |  |  |  |  |  |  |  |  |  |  |  |
| Temporary employment | -0.006* | (0.001) |  | -0.006* | (0.001) |  | -0.008* | (0.002) |  | -0.007* | (0.001) |  | -0.005* | (0.001) |
| Unemployment | -0.015* | (0.001) |  | -0.015* | (0.001) |  | -0.014* | (0.001) |  | -0.015* | (0.001) |  | -0.014* | (0.001) |
| self-employment | 0.001* | (0.000) |  | 0.001* | (0.000) |  | 0.001* | (0.000) |  | 0.001* | (0.000) |  | 0.001* | (0.000) |
| Micro-level control variables | ✓ |  |  | ✓ |  |  | ✓ |  |  | ✓ |  |  | ✓ |  |
| Period fixed effects | ✓ |  |  | ✓ |  |  | ✓ |  |  | ✓ |  |  | ✓ |  |
| Country fixed effects | ✓ |  |  | ✓ |  |  | ✓ |  |  | ✓ |  |  | ✓ |  |
| Country fixed slopes | ✓ |  |  | ✓ |  |  | ✓ |  |  | ✓ |  |  | ✓ |  |
| Macro-level variables |  |  |  |  |  |  |  |  |  |  |  |  |  |  |
| GDP per capita | -0.007 | (0.006) |  | -0.008 | (0.006) |  | -0.007 | (0.006) |  | -0.007 | (0.006) |  | -0.007 | (0.006) |
| Unemployment rate (female) | -0.010* | (0.002) |  | -0.010* | (0.002) |  | -0.010* | (0.002) |  | -0.010* | (0.002) |  | -0.010* | (0.002) |
| Temporary employment rate (female) | -0.010+ | (0.005) |  | -0.010+ | (0.005) |  | -0.010+ | (0.005) |  | -0.010+ | (0.005) |  | -0.010+ | (0.005) |
| Economic globalization | -0.005 | (0.005) |  | -0.006 | (0.005) |  | -0.005 | (0.005) |  | -0.005 | (0.005) |  | -0.005 | (0.005) |
| Female labour force participation rate | -0.008 | (0.007) |  | -0.008 | (0.007) |  | -0.009 | (0.007) |  | -0.008 | (0.007) |  | -0.009 | (0.007) |
| EPLR | -0.003 | (0.003) |  | 0.002 | (0.003) |  | -0.003 | (0.003) |  | -0.003 | (0.003) |  | -0.003 | (0.003) |
| EPLT | -0.009 | (0.005) |  | -0.009 | (0.005) |  | -0.009 | (0.008) |  | -0.008 | (0.005) |  | -0.009 | (0.005) |
| CBC | -0.006* | (0.003) |  | -0.006* | (0.003) |  | -0.006* | (0.003) |  | -0.002 | (0.004) |  | -0.006* | (0.003) |
| UB generosity | 0.001 | (0.001) |  | 0.001 | (0.001) |  | 0.001 | (0.001) |  | 0.001 | (0.001) |  | -0.002 | (0.002) |
| Temp. empl. * EPLR |  |  |  | -0.004 | (0.004) |  |  |  |  |  |  |  |  |  |
| Unempl. * EPLR |  |  |  | -0.011* | (0.004) |  |  |  |  |  |  |  |  |  |
| Temp. empl. * EPLT |  |  |  |  |  |  | 0.008 | (0.010) |  |  |  |  |  |  |
| Unempl. * EPLT |  |  |  |  |  |  | -0.003 | (0.007) |  |  |  |  |  |  |
| Temp. empl. * CBC |  |  |  |  |  |  |  |  |  | -0.005* | (0.002) |  |  |  |
| Unempl. * CBC |  |  |  |  |  |  |  |  |  | -0.005* | (0.002) |  |  |  |
| Temp. empl. * UB generosity |  |  |  |  |  |  |  |  |  |  |  |  | 0.004* | (0.001) |
| Unempl. * UB generosity |  |  |  |  |  |  |  |  |  |  |  |  | 0.005* | (0.001) |
| n (individuals) | 115,666 |  |  | 115,666 |  |  | 115,666 |  |  | 115,666 |  |  | 115,666 |  |
| N (country-rounds) | 215 |  |  | 215 |  |  | 215 |  |  | 215 |  |  | 215 |  |
| M (countries) | 26 |  |  | 26 |  |  | 26 |  |  | 26 |  |  | 26 |  |

*Note*: Significance levels: + p<0.1, * p < 0.05. Standard errors were clustered at the country level.

**Table S7. Sensitivity analysis: Results comparison of unemployment benefits’ moderating effects across different unemployment duration.**

|  | Model 1 | | Model 2 | | | Model 3 | |
| --- | --- | --- | --- | --- | --- | --- | --- |
|  | UB @ 12th  month | | UB @ 18th  month | | | UB @ 24th  month | |
| **Female** |  |  | |  |  | |  |
| Temp. empl. * UB generosity | 0.004* | | 0.004* | | | 0.007* | |
|  | (0.001) | | (0.001) | | | (0.002) | |
| Unempl. * UB generosity | 0.003* | | 0.004* | | | 0.006* | |
|  | (0.001) | | (0.001) | | | (0.001) | |
| **Male** |  |  | |  |  | |  |
| Temp. empl. * UB generosity | 0.003* | | 0.004* | | | 0.005* | |
|  | (0.001) | | (0.001) | | | (0.002) | |
| Unempl. * UB generosity | 0.004* | | 0.005* | | | 0.007* | |
|  | (0.000) | | (0.001) | | | (0.001) | |

*Note*: Models presented in this table are the same as Model 5 in Table S5 and Model 5 in Table S6. Significance levels: + p<0.1, * p < 0.05. Standard errors were clustered at the country level.

**Table S8. Overview of hypotheses and empirical supports**

| Hypotheses | Empirical supports | | |
| --- | --- | --- | --- |
|  | Female sample | Male sample | Gender difference |
| H1: Having an unstable employment situation such as temporary employment or unemployment has a negative effect on the probability of union formation among single individuals. | + | + | (H2) |
| H2: The negative effect of unstable employment situations on union formation is stronger for men than for women. |  |  | + |
| H3a: Stricter EPLR reinforces the negative effect of unstable employment situations on union formation.  H3b: The reinforcement effect of stricter EPLR is particularly strong in the negative relationship between unemployment and union formation. | –    – | /  + | (H7) |
| H4a: Stricter EPLT reinforces the negative effect of unstable employment situations on union formation.  H4b: The reinforcement effect of stricter EPLT is particularly strong in the negative relationship between unemployment and union formation. | /  + | –  – | (H8) |
| H5a: Higher CBC reinforces the negative effect of unstable employment situations on union formation.  H5b: The reinforcement effect of higher CBC is particularly strong in the negative relationship between unemployment and union formation. | /  + | +  – |  |
| H6: More generous unemployment benefits attenuate the negative effect of unstable employment situations on union formation. | + | + |  |
| H7: Stricter EPLR reinforces the negative effect of unstable employment situations on union formation especially for men. |  |  | + |
| H8: Stricter EPLT reinforces the negative effect of unstable employment situations on union formation especially for women. |  |  | + |

*Note*: + denotes empirical support, / denotes partial empirical support (only for the unemployed), – denotes no empirical support

**Fig S1. Changes in labour market regulations across country**

*Note*: The hollow dots present the initial values of regulation indicators in a specific country at the first observation (around 2010); the arrows indicate the directions and sizes of regulation changes toward the end of our observation (around 2018).

**Fig S2. Effects of temporary employment and unemployment on union formation probability by educational levels**

*Note*: Point estimates with 95% confidence intervals. The effects are calculated as probability differences with permanent employment as the reference groups. All models include control variables that are identical to those included in Table 3.

**Fig S3. Predictive probability of union formation for women by employment status**

**Fig S4. Predictive probability of union formation for men by employment status**

**Appendix A: Influential cases (outliers) analysis**

In this appendix, we checked the issue of influential cases (outliers) in multilevel analysis (Schmidt-Catran et al., 2019) and evaluated whether the estimated policy moderating effects were overrepresenting specific countries’ extreme labour market changes. To this end, Fig S5 compared our main analyses with the estimates using reduced samples, in which we excluded countries with the most extreme changes in specific labour market regulations. These outlier countries were Portugal for EPLR analyses, Lithuania for EPLT analyses, Greece for CBC analyses, and Italy for unemployment benefits analyses. Fig S5 showed that models using full samples and reduced samples produced very similar point estimates and CIs in most cases. However, noticeable differences were found in the CBC analyses, where models using reduced samples (i.e., excluding Greece) produced very wide CIs, rendering severe statistical uncertainty. Such results indicated that moderating effects of CBC found in the cFES models might overrepresent Greece’s unique experience following an extreme CBC reduction during years 2012–2016 (more than 80 percentage points reduction).

**Fig S5. Robustness analysis: Estimated policy moderating effects for samples with and without outlier countries.**

**
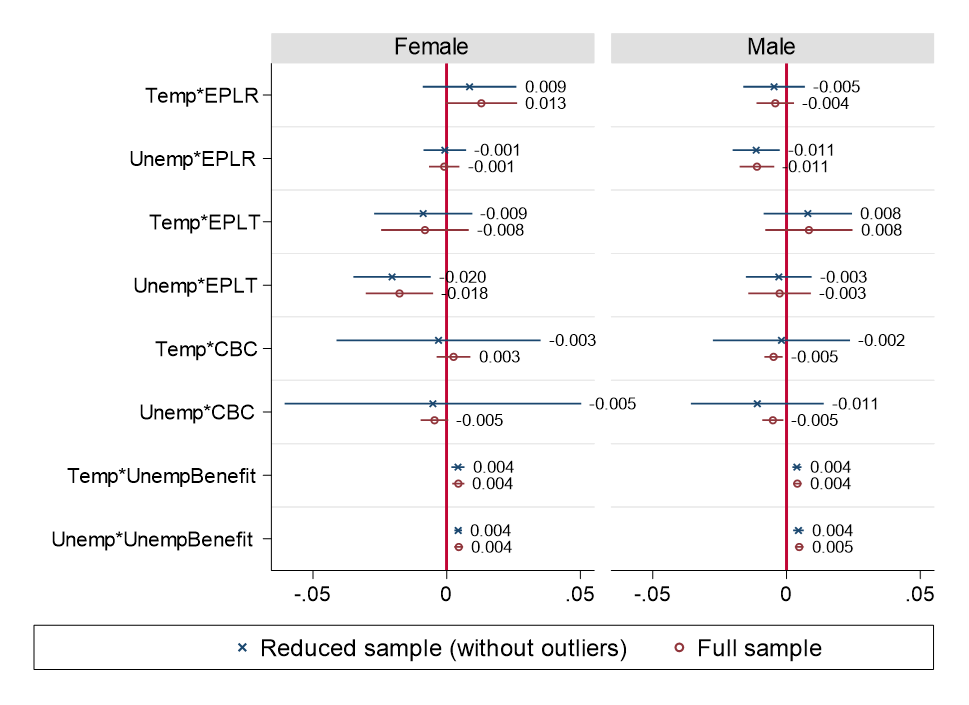
**

*Note*: Point estimates with 95% confidence intervals. All models included control variables included in Table 3. Outlier countries in the corresponding policy analyses were: (1) Portugal for EPLR analyses, (2) Lithuania for EPLT analyses, (3) Greece for CBC analyses, and (4) Italy for unemployment benefits analyses

**Appendix B: Estimation results comparison between cFES and mixed-effects models**

In this Appendix, we compared the estimation results between our cFES models and the mixed-effects models in Fig S6. In general, the two models found similar labour market moderating effects regarding the effect directions. Still, there were two incongruences worth mentioning. First, the absolute sizes of the moderating effects of EPLR and EPLT were larger in cFES models than in mixed-effects models. This pattern might be driven by the relatively stable EPLs in some countries during years 2010-2019 in our data. Because the cFES models relied on within-country changes in EPLs to estimate their moderating effects, the estimation results were sensitive and possibly inflated in the context of insufficient within-country variations. Such statistical uncertainties were reflected in the wide 95% CIs in the cFES models. Second, although the cFES models estimated positive and statistically significant moderating effects of higher unemployment benefits on the relationship between unstable employment and union formation, the mixed-effects models found nearly zero moderating effects of unemployment benefits. As discussed in the method section, the mixed-effects models could yield biased estimates if there were unobserved country-level heterogeneities influencing both the unemployment benefits and the effects of unstable employment statuses on union formation. The fact that we observed positive moderating effects of unemployment benefits only in the cFES models might indicate the existence of unobserved country-level heterogeneities that were not accounted for in the mixed-effects models, such as cultural values.

**Fig S6. Modeling results comparison between cFES models and mixed-effects models: the moderating effects of labour market regulations**

*Note*: Point estimates with 95% confidence intervals. All models included control variables included in Table 3.

**Appendix C: Case studies of labour market moderating effects**

To check the credibility of our main findings, we performed additional case studies to examine whether the policy moderating effects generalized from cross-national comparative analyses can be applied to the experiences of specific countries (see Adsera (2011) for similar applications of this methodology in family demography). To this end, we identified four representative countries and estimated the time dynamics of the effects of unstable employment situations on union formation in these countries by adding interaction terms between employment situations and years in each country models. These models included all control variables included in Table 3.

To isolate the impact of policy changes in a specific dimension, the representative countries should not have more than one substantial labour regulation reforms during the observation period. Therefore, countries that experienced the most substantial reforms in specific labour market regulations were not necessarily the most ideal case to study. For example, while Greece had the largest reduction in CBC during our observation period, it was not the most suitable for studying the influences of CBC reforms because the country also experienced remarkable increase in unemployment benefits replacement rates during the observation period (see Fig 1 for details). Instead, Finland was a suitable case to study because while Finland’s CBC rate had a remarkable increase, the other three labour market regulations remained largely unchanged in the country, making it possible to isolate the influences of CBC reforms.

**Fig S7. Effects of temporary employment and unemployment on union formation in Portugal (EPLR decreased by 0.96 in 2012-2014)**

Fig S7 presents the time dynamics of the effects of temporary employment and unemployment compared to permanent employment in Portugal, where EPLR decreased from 3.95 in 2011 to 2.99 in 2014. According to our theoretical framework, such a decrease in EPLR might attenuate the negative effects of unstable employment situations on union formation (Hypothesis 3a), especially for men (Hypothesis 7). Fig S7’s results for Portugal provided some evidence to these expectations. For men, we found that the negative effects of unstable employment on union formation became smaller during 2012–2014 comparing to the pre-reform years. For women, however, the effects of unstable employment situations on union formation became negative during the years of EPLR reduction—a pattern that contradicted Hypothesis 3a.

**Fig S8. Effects of temporary employment and unemployment on union formation in Estonia (EPLT increased by 0.75 in 2012-2013)**

To examine the moderating effects of EPLT changes, Fig S8 presented how micro-level effects of unstable employment on union formation were evolving in Estonia, where EPLT increased sharply from 2.29 in 2012 to 3.04 in 2013. Such an increase in EPLT is expected to cause a more negative effect of unstable employment situations on union formation (Hypothesis 4a), especially for women (Hypothesis 8). Results for Estonian women in Fig S8 provided some evidence to the theoretical expectations. After Estonia’s sharp increase in EPLT in 2013, the negative effect of women’s temporary employment on union transition had strengthened, and the once positive effect of women’s unemployment turned negative. However, such a dynamic effect pattern following the EPLT reform was not observed for Estonian men.

**Fig S9. Effects of temporary employment and unemployment on union formation in Finland (CBC increased by 14.1 percentage points in 2011-2014)**

As mentioned earlier, Finland was an ideal case to study the moderating effects of CBC reforms on the relationship between employment and union formation. Finland’s CBC rate increased remarkably from 77.8% in 2010 to 91.9% in 2014. Such an increase in CBC is expected to trigger a more negative effect of unstable employment situations on union formation (Hypothesis 5a). This theoretical expectation seemed to be supported by results in Fig 9, in which a downward trend toward more negative effect of unstable employment was clearly found. Nevertheless, we should also notice that the most negative effect of unstable employment on union formation was observed before the reform (i.e., 2010). It was during the years of the CBC reform that the effect became less negative or turned positive before its post-reform plunge. We therefore suggest a cautious interpretation of CBC’s moderation effect in Finland.

Finally, Italy was selected to study how unemployment benefit reforms may moderate the effect of unstable employment on union formation. During the years 2014–2015, the income replacement rate of unemployment benefits at the 18^th^ month of unemployment had significantly increased from 0 to 40 percent in Italy. In theory, such an increase in unemployment benefits might attenuate the negative influence of unstable employment on union formation (Hypothesis 6). Results in Fig S10 provided some evidence to the expectation, showing that the negative effects of unstable employment had reduced to nearly zero either during or after the unemployment benefits reform for Italian women and men.

**Fig S10. Effects of temporary employment and unemployment on union formation in Italy (UB replacement rate increased by 40 percentage points in 2014-2015)**

In summary, case studies in Appendix C provided additional supports to our theoretical hypotheses. However, due to small sample sizes for each country-rounds, the yearly effects of temporary employment and unemployment were estimated with high statistical uncertainty. Such uncertainty was reflected in the wide 95% CIs across all case studies. Rather than overinterpreting the findings, we suggest using these cases only as supplementary evidence to our main findings in the multilevel models.

**Additional References**

Adsera, A. (2011). The Interplay of Employment Uncertainty and Education in explaining Second Births in Europe. *Demographic Research*, *25*(16), 513–544. <https://doi.org/10.4054/DemRes.2011.25.16>

Schmidt-Catran, A. W., Fairbrother, M., & Andreß, H.-J. (2019). Multilevel Models for the Analysis of Comparative Survey Data: Common Problems and Some Solutions. *Kölner Zeitschrift Für Soziologie & Sozialpsychologie*, *71*, 99–128. <https://doi.org/10.1007/s11577-019-00607-9>
